# Neural Correlates of Peripheral Inflammation in Major Depressive Disorder and Their Transcriptomic Architecture, Neurochemical Basis, and Behavioral Relevance

**DOI:** 10.1002/hbm.70371

**Published:** 2025-09-27

**Authors:** Wenming Zhao, Dao‐min Zhu, Yongqi Zhang, Yu Zhang, Yuxian Shen, Yongqiang Yu, Jiajia Zhu

**Affiliations:** ^1^ Department of Radiology The First Affiliated Hospital of Anhui Medical University Hefei China; ^2^ Research Center of Clinical Medical Imaging, Anhui Province Hefei China; ^3^ Anhui Provincial Institute of Translational Medicine Hefei China; ^4^ Anhui Provincial Key Laboratory for Brain Bank Construction and Resource Utilization Hefei China; ^5^ Department of Sleep Disorders Affiliated Psychological Hospital of Anhui Medical University Hefei China; ^6^ Hefei Fourth People's Hospital Hefei China; ^7^ Anhui Mental Health Center Hefei China; ^8^ School of Basic Medical Sciences Anhui Medical University Hefei China; ^9^ Biopharmaceutical Research Institute Anhui Medical University Hefei China

**Keywords:** behavioral domain, inflammation, magnetic resonance imaging, major depressive disorder, neurotransmitter, transcriptome

## Abstract

The role of inflammation in the neuropathology of major depressive disorder (MDD) is evident. However, the neural correlates of peripheral inflammation in MDD and their transcriptomic architecture, neurochemical basis, and behavioral relevance have not been systematically investigated. We adopted functional and diffusion magnetic resonance imaging to assess gray matter function and white matter integrity, whose associations with serum C‐reactive protein (CRP) levels were explored in a large sample of MDD patients. Further, we examined the spatial relationships of the identified neural correlates of CRP with transcriptome, neurotransmitter, and behavioral domain atlases. Higher serum CRP levels were associated with local gray matter function alterations and widespread white matter integrity changes in MDD patients, but not HC. Moreover, the gray matter functional correlates of CRP in MDD were spatially correlated with functional gene categories involving inflammatory signaling pathways (macrophage activation, NF‐κB signaling, and JUN kinase activity), specific neurotransmitters (serotonin, GABA, and glutamate), and diverse behavioral domains (sensorimotor, cognition, emotion, and sleep). In addition, some neural correlates of CRP (anterior cingulate cortex function and superior corona radiata integrity) mediated the relationships of serum CRP with sustained attention and sleep structure in MDD patients. Our findings may not only confirm the role of inflammation in the neuropathology of MDD, but also inform a novel conceptualization of targeting inflammatory processes to treat this disorder.

## Graphical abstract

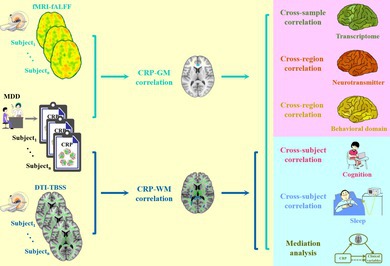

## 1 Introduction

As the single greatest cause of disability in the world, major depressive disorder (MDD) has devastating effects on all aspects of a patient's personal life and represents a major contributor to the global burden of diseases (Marx et al. 2023). The etiology of MDD is multifactorial and remains incompletely understood. It is well documented that dysregulation of the innate and adaptive immune systems occurs in MDD patients and hinders antidepressant responses (Beurel et al. 2020; Khandaker et al. 2017). Moreover, there have been attempts to elucidate the mechanisms by which the immune systems, particularly inflammation, interact with the brain to influence the risk for MDD (Miller and Raison 2015). According to the social signal transduction theory of depression, experiences of social threat and adversity upregulate components of the immune system involved in inflammation that may drive the pathogenesis of depression (Slavich and Irwin 2014). Indeed, a previous meta‐analysis showed that individuals exposed to childhood trauma had significantly elevated peripheral levels of C‐reactive protein (CRP), interleukin‐6 (IL‐6), and tumor necrosis factor‐α (TNF‐α) (Baumeister et al. 2016). A recent review also suggests that CRP and IL‐6 mediate the association between adverse childhood experiences and adult depression (Zagaria et al. 2024). These previous studies highlight the role of inflammation in the neuropathology of MDD and the therapeutic potential of targeting inflammatory processes to treat this disorder.

The level of serum CRP is a sensitive but non‐specific marker of inflammation that responds rapidly to changes in underlying inflammatory activity, making its measurement a useful tool for the detection and monitoring of many acute and chronic inflammatory conditions (Rhodes et al. 2011; Windgassen et al. 2011). A large body of cross‐sectional research has demonstrated that higher serum CRP levels are associated with MDD (Orsolini et al. 2022) as well as its core clinical features such as cognitive impairment (Dalkner et al. 2020; Mac Giollabhui et al. 2021; Zainal and Newman 2021) and sleep disturbance (Okun et al. 2009). Some longitudinal studies have also indicated that elevated serum CRP levels contribute to an increased risk of subsequent depressive symptoms (Luukinen et al. 2010; Pasco et al. 2010; Valkanova et al. 2013). These prior data have established CRP as an important inflammatory marker for MDD.

Functional magnetic resonance imaging (fMRI) is a non‐invasive neuroimaging technique to assess brain gray matter function (Biswal et al. 1995). Earlier fMRI studies have found an intimate link between peripheral CRP and gray matter dysfunction in MDD patients. For instance, a study reported that increased CRP was associated with decreased functional connectivity between the ventral striatum and the ventromedial prefrontal cortex (vmPFC), which in turn correlated with increased anhedonia in MDD patients, and increased CRP predicted decreased dorsal striatal to vmPFC and presupplementary motor area connectivity, which correlated with decreased motor speed and increased psychomotor slowing (Felger et al. 2016). Further mediation analyses revealed that these effects of CRP on functional connectivity mediated the relationships of CRP with anhedonia and motor slowing. Meanwhile, another study found that CRP was associated with reduced functional connectivity in a widely distributed network including the ventral striatum, parahippocampal gyrus/amygdala, orbitofrontal and insular cortices, and the posterior cingulate cortex in MDD patients, and these broad alterations were centralized in the vmPFC, representing a hub for the effects of inflammation on network function in the whole brain (Yin et al. 2019). Moreover, a recent study revealed that CRP was negatively correlated with functional connectivity between the left dorsal caudate putamen and the right superior frontal gyrus in MDD patients with anhedonia, and this connectivity fully mediated the relation between CRP and anhedonia (Liang et al. 2025). Despite these findings, however, little is known about the relationship between CRP and resting‐state local neural activity indexed by fMRI measures such as fractional amplitude of low‐frequency fluctuations (fALFF) (Zou et al. 2008). In addition, diffusion tensor imaging (DTI) enables the in vivo measurement of brain white matter integrity. There is strong evidence from diffusion MRI that serum levels of inflammatory markers (e.g., inflammatory cytokines and CRP) are related to white matter integrity changes in MDD patients (Lim et al. 2021; Sugimoto et al. 2018; Thomas et al. 2021). Therefore, a combined analysis of gray matter function and white matter integrity using fMRI and DTI may hold promise to achieve a more complete investigation into the neural correlates of CRP in MDD.

The current availability and refinement of whole‐brain mapping of transcriptome, neurotransmitters, and behavioral domains have opened new avenues to explore the spatial relationships between neuroimaging phenotypes and these atlases, facilitating a potentially mechanistic explanation for neuroimaging observations. By use of the Allen Human Brain Atlas (AHBA) (Hawrylycz et al. 2012; Shen et al. 2012), transcriptome‐neuroimaging spatial correlations can be carried out to examine the associations between regional gene expression profiles and neuroimaging anatomical variation patterns (Arnatkeviciute et al. 2019; Chen et al. 2022; Fang et al. 2023; Fornito et al. 2019; Li et al. 2023; Liu et al. 2022; Shen et al. 2022; Song et al. 2023; Sun et al. 2023; Xu et al. 2023; Zhang et al. 2022; Zhao, Cai, et al. 2023), followed by the performance of gene category enrichment analysis (GCEA) to determine the functional gene categories that contribute to such associations. Nonetheless, conventional GCEA is commonly affected by false‐positive biases attributable to spatial autocorrelation and gene–gene co‐expression. To mitigate this concern, a flexible ensemble‐based null model tailored to permit more valid and interpretable inference of GCEA is emerging (Fulcher et al. 2021). This methodological advance allows us to better account for the genetic architecture of neuroimaging phenotypes. With continuing improvements in nuclear imaging techniques and tracers, it is increasingly feasible to precisely and reliably measure a variety of neurotransmitter receptors and transporters that are heterogeneously distributed across the brain (Beliveau et al. 2017; Lehto et al. 2015). These neurotransmitter atlases set the stage for the study of the neurochemical basis underlying brain structure and function (Hansen et al. 2022). Neurosynth is a well‐validated and publicly available database that includes a rich range of brain activation maps pertinent to different domains of human behavior and cognition (Yarkoni et al. 2011), offering us sufficient material to attempt a thorough characterization of the behavioral relevance of neuroimaging findings. Altogether, the integration of these state‐of‐the‐art multi‐modal brain atlases with tailored analytic approaches may provide unique insights into the genetic architecture, neurochemical basis, and behavioral relevance of the neural correlates of CRP in MDD.

**FIGURE 1:**
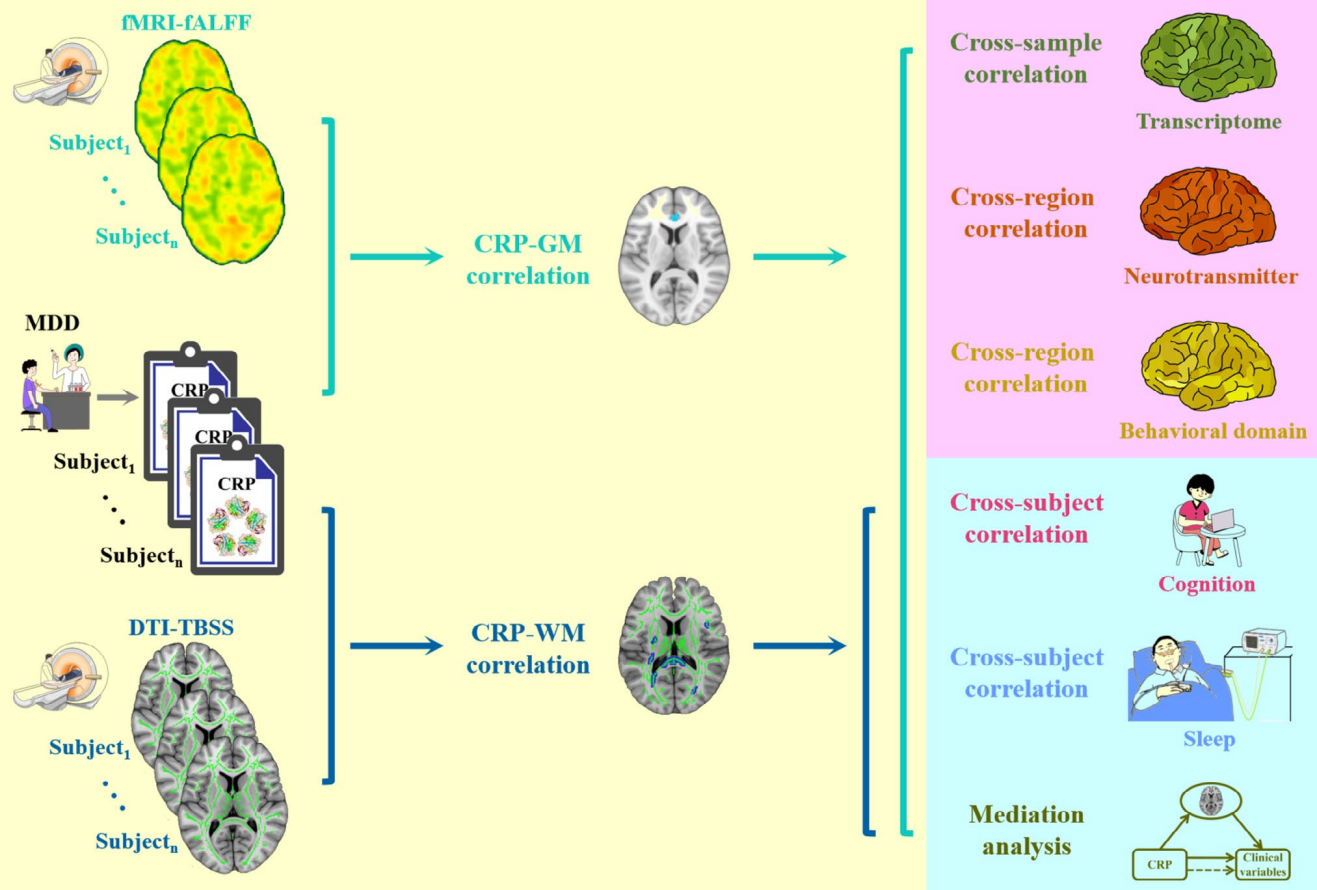
Study design and analysis pipeline. First, we adopted fMRI and DTI to assess gray matter function and white matter integrity, whose associations with serum CRP levels were investigated in a large sample of MDD patients. Second, we examined the spatial relationships of the identified neural correlates of CRP with transcriptome, neurotransmitter, and behavioral domain atlases to explore their genetic architecture, neurochemical basis, and behavioral relevance. Finally, we further investigated the correlations between CRP, neuroimaging, and clinical variables (symptom, cognition, and sleep) in MDD patients, with the aim of testing the hypothesis that neuroimaging would mediate the associations between CRP and clinical variables. CRP, C‐reactive protein; DTI, diffusion tensor imaging; fALFF, fractional amplitude of low frequency fluctuations; fMRI, functional magnetic resonance imaging; GM, gray matter; MDD, major depressive disorder; TBSS, tract‐based spatial statistics; WM, white matter.

Our goals in the present study were 3‐fold. First, we adopted fMRI and DTI to assess gray matter function and white matter integrity, whose associations with serum CRP levels were investigated in a large sample of MDD patients. Second, we examined the spatial relationships of the identified neural correlates of CRP with transcriptome, neurotransmitter, and behavioral domain atlases to explore their transcriptomic architecture, neurochemical basis, and behavioral relevance. Finally, we further investigated the correlations between CRP, neuroimaging, and clinical variables (symptom, cognition, and sleep) in MDD patients, with the aim of testing the hypothesis that neuroimaging would mediate the associations between CRP and clinical variables. A schematic overview of the study design and analysis pipeline is displayed in Figure 1.

## 2 Materials and Methods

### 2.1 Participants

The detailed information of MDD patients and HC has been described in a previous study (Zhao, Zhu, et al. 2023) and the Supporting Information. We ultimately included 238 subjects, including 120 MDD patients and 118 HC. This study was approved by the ethics committee of The First Affiliated Hospital of Anhui Medical University. After obtaining a complete description of the study, all subjects signed a written informed consent form.

### 2.2 High‐Sensitivity CRP Measurement

After abstaining from strenuous exercise for a week and fasting for an overnight period, peripheral venous blood samples (2 mL) were taken from all participants in the morning of MRI scanning and then centrifuged to generate serum that was stored at −80°C. Serum levels of high‐sensitivity CRP were measured by latex immunoturbidimetric assay using the AU5800 full‐automatic biochemical analyzer (Beckman Coulter Inc.). During the CRP measurement, two‐level quality control was performed with the coefficients of variation of the low‐level and high‐level quality control materials being 2.55%–3.90% and 3.40%–3.87%, respectively.

### 2.3 Cognitive Assessment

There is consistent evidence that sustained attention deficit is a typical feature of depression (Rock et al. 2014), such that we examined this cognitive function here. A clinical psychiatrist trained in neuropsychological testing conducted a computerized version of the Continuous Performance Task‐Identical Pairs (CPT‐IP) (Cornblatt et al. 1988) to measure sustained attention for all subjects. The detailed steps are presented in the Supporting Information.

### 2.4 Polysomnography Examination

To assess objective sleep, overnight polysomnography monitoring was performed on 118 MDD patients using an Embla N7000 instrument (New York, NY, United States). During the polysomnography examination, neurophysiological variables (electroencephalogram, electrooculogram, chin electromyogram, and lower extremity movement), cardiorespiratory variables (electrocardiogram, thoracic and abdominal movements, oxygen saturation, and oronasal flow), and other variables (e.g., body position) were recorded and analyzed. Sleep stages including rapid eye movement (REM) and non‐REM (N1, N2, and N3) were scored and double‐checked based on the American Academy of Sleep Medicine (AASM) criteria (Iber et al. 2007). In our design, the following variables were calculated: total time in bed (TIB), total sleep time (TST), sleep efficiency (ratio of TST to TIB), and percentage of total sleep time spent in each sleep stage (N1%, N2%, N3%, and REM%).

### 2.5 MRI Data Acquisition and Processing

MRI data were acquired on a 3.0‐T MR scanner (Discovery MR750w, General Electric, Milwaukee, WI, USA). The image acquisition parameters as well as the detailed processing steps of the fMRI and DTI data are described in the Supporting Information.

### 2.6 Gray Matter Function Analysis

Gray matter function was assessed by fALFF that is calculated in the following way. The BOLD time course of each voxel obtained from the preprocessed fMRI data was transformed to a frequency domain via a Fast Fourier Transform and then the power spectrum was obtained. fALFF was defined as the ratio of the power spectrum in the low‐frequency band (0.01–0.1 Hz) to that in the entire frequency range (Zou et al. 2008). For the purpose of standardization, the fALFF value of each voxel was divided by the global mean fALFF value, yielding a standardized fALFF map per subject.

We tested the voxel‐wise correlations between CRP and fALFF in MDD patients and HC, separately. A multiple regression model in Statistical Parametric Mapping (SPM12, http://www.fil.ion.ucl.ac.uk/spm) was used to identify any voxels in the fALFF maps that exhibited a significant association with CRP while controlling for age, sex, education, and frame‐wise displacement (FD). The voxel‐based statistical analysis yielded a *t* map, representing correlations between CRP and fALFF. Multiple comparisons were corrected using the cluster‐level family‐wise error (FWE) method, resulting in a cluster defining threshold of *p* = 0.001 and a corrected cluster significance of *p* < 0.05. Then, the fALFF of each cluster with a significant correlation with CRP was extracted and used for region of interest (ROI)‐based analyses. In case of significant correlations identified in MDD patients or HC, we further examined whether there were significant group differences in the correlations. That is, the correlation coefficients were transformed into Fisher's *Z* scores and then compared between MDD patients and HC.

### 2.7 Spatial Correlation With Transcriptome

Brain transcriptomic data were obtained from the AHBA dataset (http://www.brain‐map.org) (Hawrylycz et al. 2015; Hawrylycz et al. 2012). Expression levels of more than 20,000 genes at 3702 spatially distinct tissue samples were measured in six human donor brains (Table S1 in the Supporting Information). A newly proposed pipeline was employed to process the transcriptomic data (Arnatkeviciute et al. 2019). The detailed processing steps are described in the Supporting Information. After these processing steps, we obtained normalized expression data of 5013 genes for 871 tissue samples, resulting in a sample × gene matrix of 871 × 5013.

We adopted transcriptome‐neuroimaging spatial correlation and the newly developed ensemble‐based GCEA to investigate the transcriptomic architecture underlying the neural correlates of CRP. The detailed processing steps are described in the Supporting Information.

### 2.8 Spatial Correlation With Neurotransmitters

JuSpace (https://github.com/juryxy/JuSpace) is a useful tool allowing for spatial correlation analyses between cross‐modal neuroimaging data (Dukart et al. 2021). Here, to determine the neurochemical basis underlying the neural correlates of CRP, we adopted JuSpace to investigate the spatial correlations of the *t* map with nuclear imaging derived measures covering various neurotransmitter systems including dopamine, serotonin, glutamate, GABA, acetylcholine, opioid, cannabinoid, noradrenaline, and fluorodopa (Table S2 in the Supporting Information). Specifically, Pearson correlation coefficients between the *t* map and these neurotransmitter maps were calculated across 246 cerebral regions derived from the Human Brainnetome Atlas (Fan et al. 2016) while adjusting for spatial autocorrelation and partial volume with the gray matter probability map. Exact *p* values were computed using spatial permutation‐based null maps with 5000 permutations. Multiple comparisons were adjusted by the Benjamini and Hochberg method for false discovery rate (FDR) with a corrected significance level of *p* < 0.05.

### 2.9 Spatial Correlation With Behavioral Domains

To capture the behavioral relevance of the neural correlates of CRP, we examined their spatial correlations with behavioral domains from the Neurosynth (http://www.neurosynth.org), a well‐validated and publicly available platform for meta‐analysis of neuroimaging literature (Yarkoni et al. 2011). The Neurosynth database provides activation (*z*‐statistics) maps of a wide range of behavioral terms that describe nearly all aspects of human behavior. For ease of interpretability, we selected the 50 most relevant behavioral terms *ad hoc* and focused our analysis on them (Supplementary File 1). Likewise, we used JuSpace to calculate cross‐region Pearson correlation coefficients between the *t* map and the 50 Neurosynth maps while controlling for spatial autocorrelation and partial volume. Exact permutation‐based *p* values were computed using the above‐described method, and a FDR corrected *p* < 0.05 was considered significant.

### 2.10 White Matter Integrity Analysis

White matter integrity was assessed by diffusion parameters including fractional anisotropy (FA), axial diffusivity (AD), radial diffusivity (RD), and mean diffusivity (MD). Voxel‐wise statistical analyses of these diffusion parameters were performed using a tract‐based spatial statistics (TBSS) pipeline (Smith et al. 2006). Briefly, individual FA images were initially transformed to the MNI space (Mazziotta et al. 1995) by using the FMRIB Non‐linear Imaging Registration Tool. After transformation into the MNI space, a mean FA image was created and thinned to generate a common white matter skeleton. Then, individual transformed FA, AD, RD, and MD images were projected onto this white matter skeleton.

Voxel‐wise correlations between CRP and these diffusion parameters were conducted using a permutation‐based inference tool for nonparametric statistics in MDD patients and HC, respectively. For the correlation analyses, several nuisance covariates (age, sex, and education) were included, and the number of permutations was set at 5000 (Nichols and Holmes 2002). Multiple comparison correction was carried out by using a combination of threshold‐free cluster enhancement (TFCE) (Smith and Nichols 2009) and FWE, resulting in a significance threshold of corrected *p* < 0.05. Then, the diffusion parameters of each cluster with significant correlation with CRP were extracted and used for ROI‐based analyses. In case of significant correlations identified in MDD patients or HC, we further transformed the correlation coefficients into Fisher's *Z* scores and then examined whether there were significant group differences in the correlations.

### 2.11 Correlations Between CRP, Neuroimaging, and Clinical Variables

The neuroimaging value (fALFF and diffusion parameters) of each cluster with significant correlation with CRP was extracted and used for ROI‐based correlation analyses with clinical variables (symptom, cognition, and sleep) in MDD patients. For these ROI‐based analyses, several nuisance covariates (fALFF: age, sex, education, and FD; diffusion parameters: age, sex, and education) were considered, and multiple comparisons were adjusted by the FDR method. Furthermore, we tested the mediation model where neuroimaging mediated the associations between CRP and clinical variables using the PROCESS macro (http://www.processmacro.org/) (Hayes 2009, 2014). The same nuisance covariates were considered in the mediation analyses. Based on 5000 bootstrap realizations, the significance of mediation effects was determined by the bootstrap 95% confidence interval (CI) in the way a significant indirect effect is indicated when the bootstrap 95% CI does not include zero.

### 2.12 Statistical Analysis

Demographic and clinical data were analyzed using the SPSS 23.0 software package (SPSS, Chicago, IL, USA). Two‐sample *t*‐tests were utilized to compare MDD patients and HC in CRP, age, education, body mass index (BMI), FD, HAMD, HAMA, and CPT‐IP scores. Pearson Chi‐square test was adopted to examine group difference in sex. A threshold of *p* < 0.05 was considered statistically significant (two‐sided). In addition, we tested the associations between CRP and clinical variables using partial correlation analyses controlling for age, sex, and education in the patient and control groups, respectively. Multiple comparison correction was performed using the FDR method.

### 2.13 Sensitivity Analysis

First, prior research has reported significant effects of BMI on CRP in patients with MDD (Chamberlain et al. 2019), so we repeated the analyses while considering BMI as an additional nuisance covariate to control for its potential impact. Second, our MDD patients were receiving antidepressant medications and had different illness durations, which may affect our interpretation. To exclude their influences, we included antidepressant types and illness durations as additional nuisance covariates in the analyses. Third, to better align findings across imaging modalities, we repeated spatial correlation with transcriptome using partial least squares (PLS) regression in a cross‐region manner according to the Human Brainnetome Atlas. The detailed processing steps are described in the Supporting Information.

## 3 Results

### 3.1 Demographic and Clinical Characteristics

Demographic and clinical characteristics of the participants are shown in Table 1. Briefly, there were no significant differences in CRP, age, sex, BMI, or FD between the patient and control groups. However, MDD patients showed lower education, CPT‐IP‐2, CPT‐IP‐3, and CPT‐IP‐4, and higher HAMD and HAMA than HC.

**TABLE 1:** Demographic and clinical characteristics of the sample.

Characteristic	MDD (*N* = 120)	HC (*N* = 118)	Statistic	*p*
Sex (female/male)	81/39	79/39	χ^2^ = 0.008	0.928
Age (years)	42.89 ± 11.03 (18–62)	43.77 ± 13.89 (21–65)	*t* = −0.540	0.590
Education (years)	8.87 ± 3.66 (0–16)	11.64 ± 4.67 (0–20)	*t* = −5.091	< 0.001
CRP (mg/L)	0.98 ± 1.27 (0.02–6.39)	1.10 ± 0.99 (0.20–5.00)	*t* = −0.770	0.442
BMI (kg/m^2^)	22.97 ± 3.80 (13.93–34.60)	23.17 ± 2.73 (15.98–31.56)	*t* = −0.474	0.636
HAMD	28.84 ± 11.42 (1–55)	1.13 ± 2.55 (0–19)	*t* = 25.971	< 0.001
HAMA	19.76 ± 7.33 (2–35)	1.43 ± 2.88 (0–21)	*t* = 25.471	< 0.001
CPT‐IP‐2	2.22 ± 1.03 (−0.12–4.24)	3.17 ± 0.97 (0.27–4.24)	*t* = −7.350	< 0.001
CPT‐IP‐3	1.67 ± 0.95 (−0.26–4.24)	2.45 ± 1.11 (−0.11–4.24)	*t* = −5.769	< 0.001
CPT‐IP‐4	0.92 ± 0.73 (−0.41–3.12)	1.36 ± 0.86 (−0.44–3.62)	*t* = −4.240	< 0.001
FD (mm)	0.13 ± 0.09 (0.04–0.60)	0.14 ± 0.08 (0.05–0.40)	*t* = −1.084	0.279
Illness duration (months)	63.01 ± 71.90 (0.30–306)	—	—	—
PSQI^a^	12.98 ± 4.83 (1–21)			
Polysomnography^b^				
TIB (min)	512.92 ± 46.36 (408.60–681.50)	—	—	—
TST (min)	442.61 ± 56.67 (305–615.50)	—	—	—
Sleep efficiency (%)	86.39 ± 8.63 (51.69–99.42)	—	—	—
N1%	16.39 ± 9.75 (2.19–61.80)	—	—	—
N2%	64.78 ± 12.84 (25.77–92.65)	—	—	—
N3%	6.15 ± 7.29 (0–32.88)	—	—	—
REM%	12.67 ± 6.20 (0.25–33.12)	—	—	—
Antidepressant medication (number of patients)				
SSRIs	79	—	—	—
SNRIs	34	—	—	—
NaSSA	7	—	—	—

*Note:* Except for sex designation, data are expressed as means ± standard deviations. Numbers in parentheses are the range.

Abbreviations: BMI, body mass index; CPT‐IP, Continuous Performance Task‐Identical Pairs; CRP, C‐reactive protein; FD, frame‐wise displacement; HAMA, Hamilton Rating Scale for Anxiety; HAMD, Hamilton Rating Scale for Depression; HC, healthy controls; MDD, major depressive disorder; NaSSA, noradrenergic and specific serotonergic antidepressant; PSQI, Pittsburgh Sleep Quality Index; REM, rapid eye movement; SNRIs, serotonin norepinephrine reuptake inhibitors; SSRIs, selective serotonin reuptake inhibitors; TIB, total time in bed; TST, total sleep time.

^a^ The data are available for 119 from 120 patients.

^b^ The data are available for 118 of 120 patients.

There were no significant correlations of CRP with symptom (HAMD and HAMA), cognition (CPT‐IP scores), or sleep (PSQI and polysomnography parameters) in MDD patients (Table S3 in the Supporting Information). In addition, no significant correlations were observed between CRP and cognition in HC.

### 3.2 Associations Between CRP and Gray Matter Function

**FIGURE 2:**
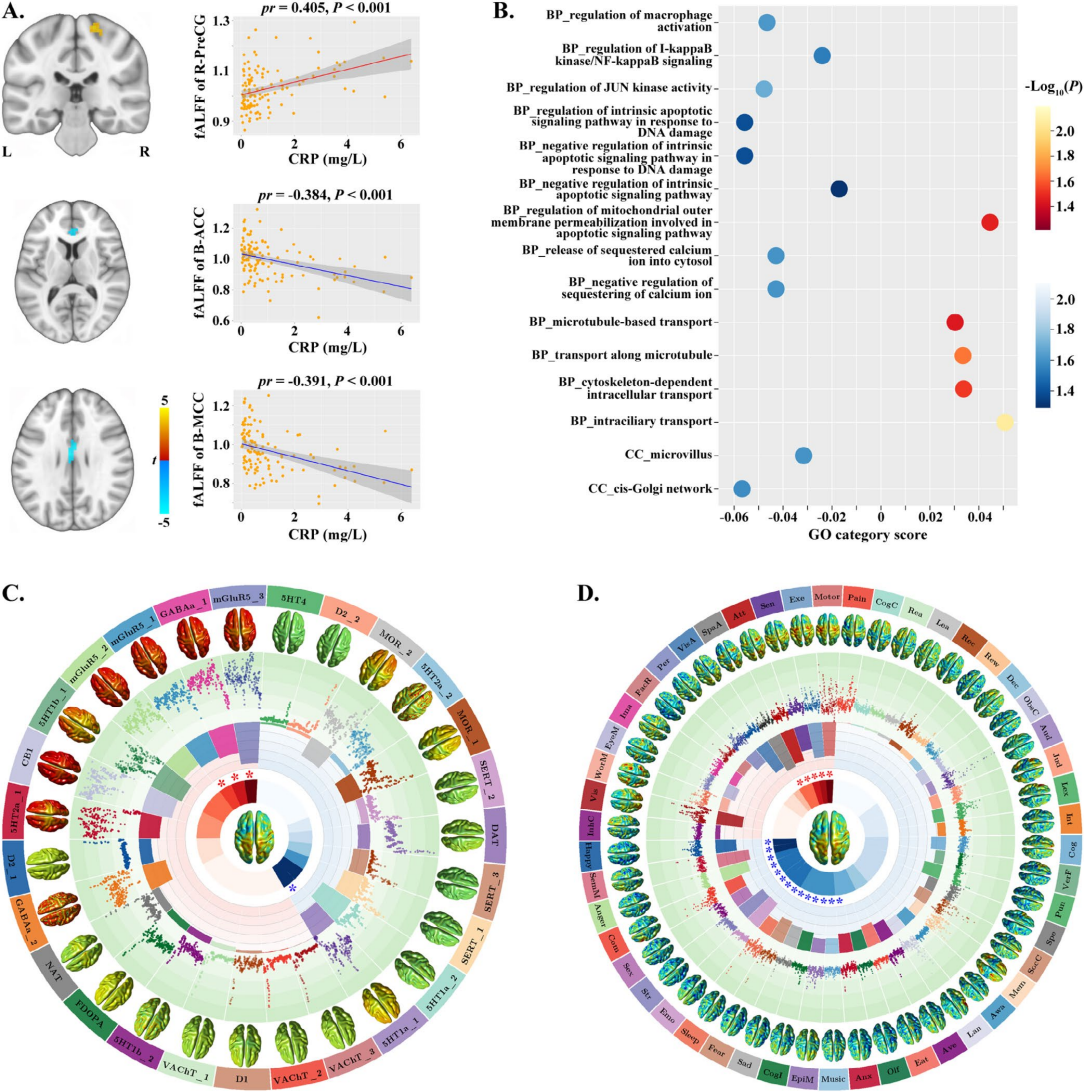
Associations between CRP and gray matter function in MDD patients. (A) On the left, the voxel‐wise correlation analysis revealed that CRP was correlated with fALFF of some gray matter regions; on the right, scatter plots show these correlations. (B) Gene categories related to the neural correlates of CRP. The warm color represents positive correlations and the cold color denotes negative correlations. (C) Neurotransmitters related to the neural correlates of CRP. The outermost ring displays the names and maps of 27 neurotransmitter receptors/transporters. The second circle displays the neurotransmitter values across 246 cerebral regions. The third circle displays the cross‐region Pearson correlation coefficients between these neurotransmitter maps and the neural correlates of CRP; the light red background represents positive correlations and the light blue background represents negative correlations. The innermost ring displays the permutation‐based statistical significance of the spatial correlations, that is, −Log_10_(*P*); *p < 0.05, FDR corrected. The *t* map for the correlations between CRP and fALFF lies in the center. (D) Behavioral domains related to the neural correlates of CRP. The outermost ring displays the names and activation maps of 50 behavioral terms from the Neurosynth. The same descriptions apply to the other circles. 5HT, 5‐hydroxytryptamine; ACC, anterior cingulate cortex; Anx, anxiety; Att, attention; Aud, auditory; Ave, aversive; Awa, awareness; B, bilateral; BP, biological process; CB, cannabinoid; CC, cellular component; Cog, cognition; CogC, cognitive control; CogI, cognitive impairment; Com, comprehension; CRP, C‐reactive protein; D, dopamine; DAT, dopamine transporter; Dec, decision; Eat, eating; Emo, emotion; EpiM, episodic memory; Exe, execution; EyeM, eye movement; FacR, face recognition; fALFF, fractional amplitude of low frequency fluctuations; FDOPA, fluorodopa; FDR, false discovery rate; GABAa, gamma‐aminobutyric acid a; GO, Gene Ontology; Ima, imagine; InhC, inhibitory control; Int, interoceptive; Jud, judgment; L, left; Lan, language; Lea, learning; Lex, lexical; MCC, middle cingulate cortex; MDD, major depressive disorder; Mem, memory; mGluR, metabotropic glutamate receptor; MOR, mu opioid receptor; NAT, noradrenaline transporter; ObsC, obsessive compulsive; Olf, olfactory; Per, perception; *pr*, partial correlation coefficient; PreCG, precentral gyrus; Pun, punishment; R, right; Rea, reasoning; Rec, recognition; Rew, rewarding; SemM, semantic memory; Sen, sensory; SERT, serotonin transporter; Sex, sexual; SocC, social cognition; SpaA, spatial attention; Spe, speech; Str, stress; VAChT, vesicular acetylcholine transporter; VerF, verbal fluency; Vis, visual; VisA, visual attention; WorM, working memory.

The voxel‐wise correlation analysis revealed that CRP was positively correlated with fALFF of the right precentral gyrus (PreCG, cluster size = 29 voxels, peak MNI coordinate x/y/z = 18/−24/66, peak *t* = 4.640), and negatively correlated with fALFF of the bilateral anterior cingulate cortex (ACC, cluster size = 28 voxels, peak MNI coordinate x/y/z = 3/36/6, peak *t* = −4.240) and bilateral middle cingulate cortex (MCC, cluster size = 27 voxels, peak MNI coordinate x/y/z = −3/−9/27, peak *t* = −4.498) in MDD patients (cluster‐level *p* < 0.05, FWE corrected) (Figure 2A). However, no significant correlations were observed between CRP and fALFF in HC (cluster‐level *p* > 0.05, FWE corrected). Notably, the significant correlations between CRP and fALFF observed in MDD patients were non‐significant in HC, with the correlation coefficients showing significant or marginally significant group differences (Table S4 in the Supporting Information).

### 3.3 Gene Categories Related to the Neural Correlates of CRP

A combination of transcriptome‐neuroimaging spatial correlation and the ensemble‐based GCEA revealed that the above‐identified neural correlates of CRP were spatially correlated with gene expression of GO categories primarily involving inflammatory signaling pathways (e.g., macrophage activation, NF‐κB signaling, and JUN kinase activity), apoptotic signaling pathway, calcium ion regulation, transport processes, and cellular components (e.g., microvillus and cis‐Golgi network) (spatially constrained permutation‐based *p* < 0.05) (Figure 2B and Supplementary File 2).

### 3.4 Neurotransmitters Related to the Neural Correlates of CRP

Cross‐region spatial correlation analyses demonstrated significant associations between the neural correlates of CRP and specific neurotransmitters (permutation‐based *p* < 0.05, FDR corrected). Briefly, the neural correlates of CRP were negatively associated with serotonin (5HT1a_1: *r* = −0.172, *p* = 0.007) and positively associated with GABA (GABAa_1: *r* = 0.191, *p* = 0.002) and glutamate (mGluR5_1: *r* = 0.179, *p* = 0.005; mGluR5_3: *r* = 0.269, *p* < 0.001) (Figure 2C and Supplementary File 3).

### 3.5 Behavioral Domains Related to the Neural Correlates of CRP

By linking the neural correlates of CRP with behavioral domains from the Neurosynth, we found that the neural correlates of CRP were spatially associated with a wide range of behavioral domains including sensorimotor (e.g., “motor” and “sensory”), cognition (e.g., “execution,” “attention,” “spatial attention,” “semantic memory,” “comprehension,” “cognitive impairment,” and “episodic memory”), emotion (e.g., “happy,” “anger,” “emotion,” “fear,” “sad,” and “anxiety”), and sleep (*p* < 0.05, FDR corrected) (Figure 2D and Supplementary File 1).

### 3.6 Associations Between CRP and White Matter Integrity

**FIGURE 3:**
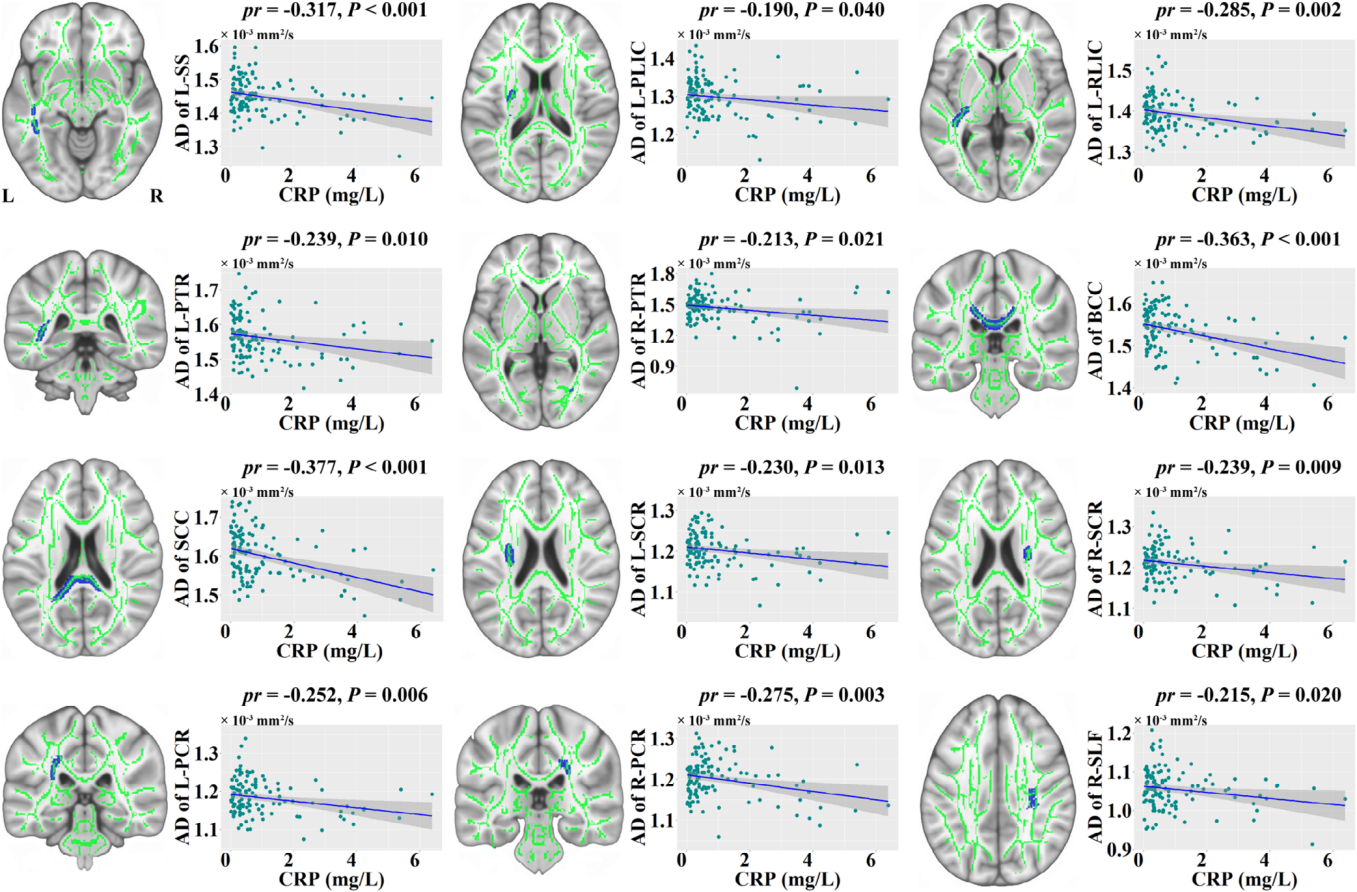
Associations between CRP and white matter integrity in MDD patients. The TBSS demonstrated that CRP was negatively correlated with AD of some white matter fiber tracts. Scatter plots show the corresponding correlations between CRP and AD. AD, axial diffusivity; BCC, body of corpus callosum; CRP, C‐reactive protein; L, left; MDD, major depressive disorder; PCR, posterior corona radiata; PLIC, posterior limb of internal capsule; *pr*, partial correlation coefficient; PTR, posterior thalamic radiation; R, right; RLIC, retrolenticular part of internal capsule; SCC, splenium of corpus callosum; SCR, superior corona radiata; SLF, superior longitudinal fasciculus; SS, sagittal stratum; TBSS, tract‐based spatial statistics.

The TBSS demonstrated that CRP was negatively correlated with AD of the left sagittal stratum, left posterior limb of internal capsule, left retrolenticular part of internal capsule, bilateral posterior thalamic radiation, body and splenium of corpus callosum, bilateral superior corona radiata, bilateral posterior corona radiata, and right superior longitudinal fasciculus in MDD patients (*p* < 0.05, TFCE‐FWE corrected) (Figure 3). However, no significant correlations were observed between CRP and any diffusion parameters in HC (*p* > 0.05, TFCE‐FWE corrected). Of note, the significant correlations between CRP and AD seen in MDD patients were non‐significant or showed opposite directions in HC, with most of the correlation coefficients showing significant or marginally significant group differences (Table S4 in the Supporting Information).

### 3.7 Associations Between CRP, Neuroimaging, and Clinical Variables

**FIGURE 4:**
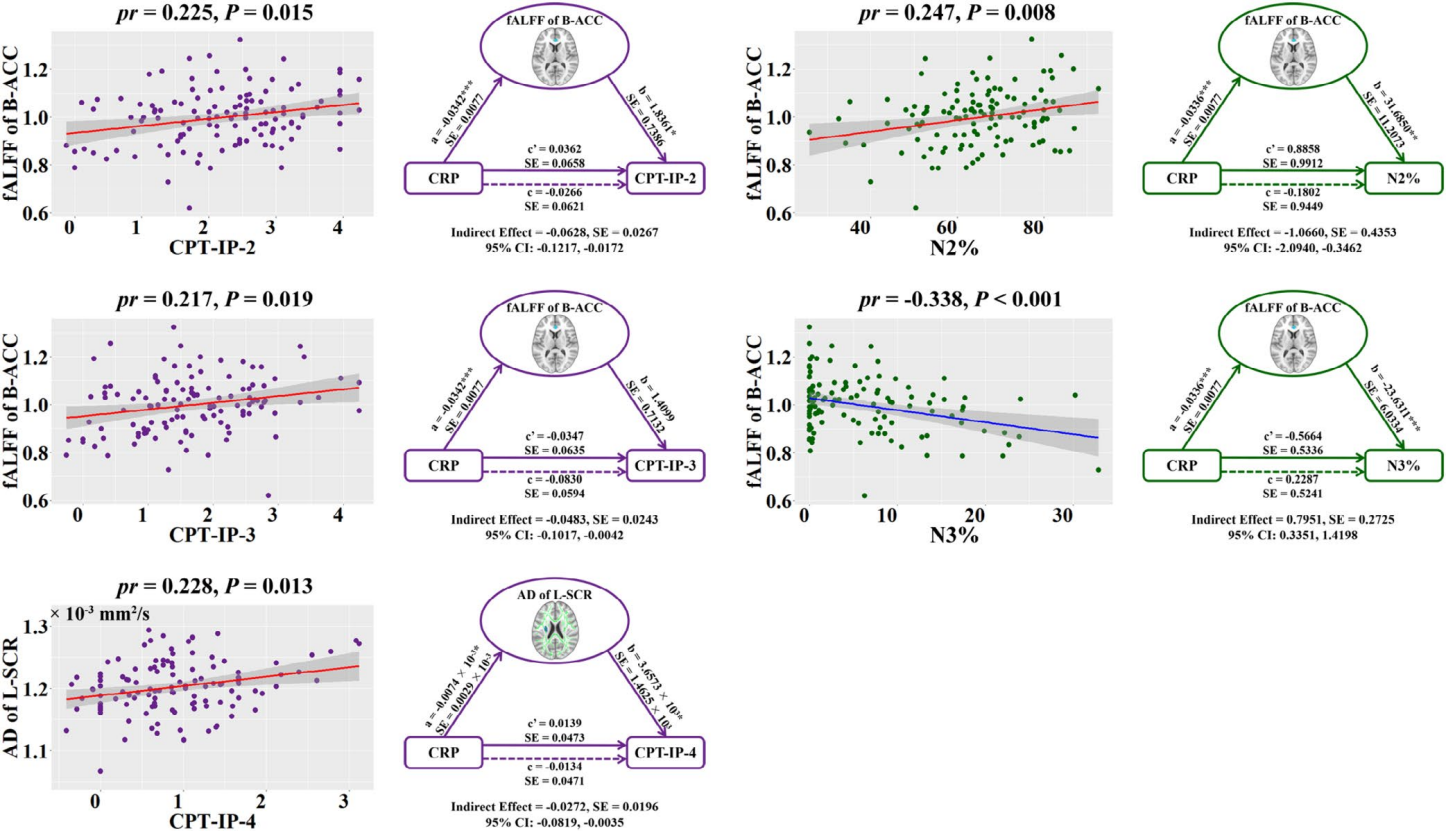
Associations between CRP, neuroimaging and clinical variables in MDD patients. On the left, scatter plots of the correlations between CRP‐related neuroimaging measures and clinical variables. On the right, graphical representation of the mediation analyses between CRP and clinical variables with the neuroimaging measures as the mediators: Estimates of the indirect (a × b), direct (c′), and total (c) effects. All paths are reported as unstandardized ordinary least squares regression coefficients. *p < 0.05; **p < 0.01; ***p < 0.001. ACC, anterior cingulate cortex; AD, axial diffusivity; B, bilateral; CI, confidence interval; CPT‐IP, Continuous Performance Task‐Identical Pairs; CRP, C‐reactive protein; fALFF, fractional amplitude of low‐frequency fluctuations; L, left; MDD, major depressive disorder; *pr*, partial correlation coefficient; SCR, superior corona radiata; SE, standard error.

The neuroimaging measures in relation to CRP were also found to be associated with clinical variables in MDD patients (*p* < 0.05, FDR corrected) (Figure 4 and Tables S5 and S6 in the Supporting Information). With respect to cognition, we found significant positive correlations between fALFF of the bilateral ACC and CPT‐IP‐2 as well as CPT‐IP‐3, and between AD of the left superior corona radiata and CPT‐IP‐4. In regard to sleep, fALFF of the bilateral ACC was positively correlated with N2% and negatively correlated with N3%. Further mediation analyses showed that the neuroimaging measures mediated the relationships between CRP and these clinical variables in MDD patients (Figure 4). That said, fALFF of the bilateral ACC significantly mediated the associations of CRP with CPT‐IP‐2, CPT‐IP‐3, N2%, and N3%; AD of the left superior corona radiata significantly mediated the association between CRP and CPT‐IP‐4.

### 3.8 Sensitivity Analysis

After additionally adjusting for BMI, the correlations between CRP and neuroimaging measures, the correlations between neuroimaging measures and clinical variables, and the mediation results were reproduced (Tables S7–S9 in the Supporting Information). Likewise, after controlling for antidepressant types and illness duration, the correlations between CRP and neuroimaging measures, the correlations between neuroimaging measures and clinical variables, and the mediation results were preserved (Tables S10–S12 in the Supporting Information). Finally, PLS regression identified the first PLS component where the weighted sum of gene expression (gene scores) was most strongly correlated with the neural correlates of CRP (*r* = 0.483) (Figure S1A in the Supporting Information), which was significantly greater than expected by chance (*P*_perm_ = 0.011) (Figure S1B in the Supporting Information). All the 5013 genes were ranked by their contributions to the correlation (i.e., gene loadings) (Figure S1C in the Supporting Information and Supplementary File 4). 698 PLS+ genes (the top 25% of genes with positive loadings) and 555 PLS‐ genes (the top 25% of genes with negative loadings) were defined as strongly contributing genes. The functional annotation found that PLS+ and PLS‐ genes were mainly enriched in inflammatory signaling pathways (e.g., regulation of macrophage proliferation, regulation of MAPK cascade), transport processes, calcium ion regulation, neurotransmitter secretion, neurons generation and differentiation, cognition, and cellular components (e.g., synapse, neuron projection, axon, dendrite, neuronal cell body, and I‐kappaB/NF‐kappaB complex) (Figure S2 in the Supporting Information).

## 4 Discussion

There were three main findings in the current study. First, we found that higher serum CRP levels were associated with local gray matter function alterations and widespread white matter integrity changes in MDD patients, but not HC. Second, the gray matter functional correlates of CRP in MDD were spatially correlated with functional gene categories involving inflammatory signaling pathways (macrophage activation, NF‐κB signaling, and JUN kinase activity), specific neurotransmitters (serotonin, GABA, and glutamate), and diverse behavioral domains (sensorimotor, cognition, emotion, and sleep). Finally, some neural correlates of CRP (ACC function and superior corona radiata integrity) mediated the relationships of serum CRP levels with sustained attention and sleep structure in MDD patients.

Our data showed that there was no difference in serum CRP levels between MDD patients and HC, which appears at odds with most previous reports of higher serum CRP in MDD patients than HC (Aruldass et al. 2021; Burrows et al. 2021; Kitzbichler et al. 2021; Opel et al. 2019). Sample heterogeneity in age, BMI, medication, illness duration, recent infection, or chronic inflammatory condition is a highly likely source of the result discrepancy. Despite the comparable serum CRP levels, the associations between CRP and neuroimaging measures were present in the patients but not the controls, suggesting that such inflammation–brain associations may occur in the context of MDD. This may be explained by the evidence that MDD‐induced neurovascular pathology alters blood–brain barrier integrity, promoting infiltration of circulating inflammatory markers into brain parenchyma and subsequent neurobiological changes (Dudek et al. 2020; Menard et al. 2017).

Numerous human neuroimaging studies have demonstrated abnormal ACC functions (e.g., functional connectivity, synaptic density, and microglial activation) in depressed patients (Holmes et al. 2019; Peng et al. 2021; Setiawan et al. 2015; Zheng et al. 2018). There is also empirical evidence that CRP is negatively correlated with cortical thickness (van Velzen et al. 2017) and brain activation during reward anticipation (Liu et al. 2020) in the ACC in MDD patients. Moreover, animal research has indicated that early‐life inflammation causes dysregulation of microglial engulfment capacity in the ACC, thus promoting the development of depression‐like symptoms during adolescence (Cao et al. 2021). Furthermore, the ACC is one of the nodes in the neural circuit involved in the bottom‐up process of interoception (Sugawara et al. 2024). Interoceptive signaling of inflammation plays a role in human depression (Savitz and Harrison 2018). Findings across neuroimaging modalities have consistently shown that inflammatory stimuli disrupt circuits and networks involved in motivation and motor activity, interoceptive and emotional processing (Goldsmith et al. 2023). Thus, these earlier reports, taken with our observation of an association between higher CRP and lower fALFF of the ACC in the patients, support the notion that inflammation‐related changes in the ACC may contribute to the pathophysiology of MDD. In addition, we found that CRP was related to fALFF of the PreCG and MCC in the patients, which suggests that the two regions might also play a role in the relationship between inflammation and MDD, complementing and extending extant literature.

Likewise, we observed that elevated CRP levels were associated with widespread white matter integrity changes in MDD patients. The affected white matter fiber tracts have been frequently reported to be compromised in MDD in prior investigations (Chen et al. 2016; Jiang et al. 2017; van Velzen et al. 2019). Moreover, the relationships between serum inflammatory markers (e.g., inflammatory cytokines and CRP) and white matter integrity in these fiber tracts have been well established in MDD patients (Lim et al. 2021; Sugimoto et al. 2018; Thomas et al. 2021). Among these studies, the most consistent finding has been the impact on the corpus callosum that interconnects the bilateral cerebral hemispheres to integrate motor, perceptual, cognitive, and emotional functions (Gazzaniga 2000; Quigley et al. 2003), making it a candidate biomarker of brain inflammation in MDD. Alternatively, the widespread nature of the affected white matter regions raises the possibility that MDD‐related systemic inflammation may influence white matter microstructure in a global and non‐specific manner.

Spatial correlation analyses demonstrated that the neural correlates of CRP in MDD were associated with functional gene categories involving inflammatory signaling pathways (macrophage activation, NF‐κB signaling, and JUN kinase activity), specific neurotransmitters (serotonin, GABA, and glutamate), and diverse behavioral domains (sensorimotor, cognition, emotion, and sleep). As tissue‐specific macrophages in the brain, microglia play a key role in neuroinflammation (Guo et al. 2020). Resident microglia become activated towards the pro‐inflammatory phenotype or the anti‐inflammatory phenotype during neuroinflammation. Abnormal communication between activated microglia and neurons might be linked to the pathogenesis of depression. NF‐κB has been implicated in coordinating the expression of a wide variety of genes that control the activation and regulation of key molecules that are associated with inflammation (Chen et al. 2012; Li and Verma 2002). JUN N‐terminal kinase (JNK) is a stress‐activated member of the mitogen‐activated protein kinase (MAPK) family, and its activation has also been strongly involved in inflammatory responses (Gao and Ji 2008; Vasilevskaya and O'Dwyer 2003). Evidence is now emerging that NF‐κB and JNK pathways may serve as important inflammatory mechanisms underlying the neuropathology in MDD (Caviedes et al. 2017; Eyre and Baune 2012; Hollos et al. 2018). In terms of neurotransmitters, it has been theorized that dysfunctional serotonin, GABA, and glutamate systems are characteristic of depression (Wang et al. 2021; Yohn et al. 2017). One potential pathway by which alterations in these transmitter systems may occur in MDD involves inflammation. That said, inflammatory responses can influence the metabolism of these neurotransmitters through effects on astrocytes and microglia, which can ultimately affect neural circuits to alter behavior, giving rise to depressive symptoms (Miller and Raison 2015). Extensive research has demonstrated deficits in sensorimotor (Malhi and Mann 2018), cognition (Rock et al. 2014), emotion (Rottenberg 2017), and sleep (Riemann et al. 2020) in patients suffering from MDD. Associations between CRP and these behavioral deficits have also been reported in depressed patients (Chamberlain et al. 2019; Dalkner et al. 2020; Mac Giollabhui et al. 2021; Milaneschi et al. 2021; Prather et al. 2015; Zainal and Newman 2021). Our finding of spatial correlations between the neural correlates of CRP and these behavioral domains from the Neurosynth corroborates the concept that inflammation might contribute to a broad range of behavioral deficits in MDD from the neurobiological perspective.

Our further mediation analyses revealed that some neural correlates of CRP (ACC function and superior corona radiata integrity) mediated the relationships of serum CRP levels with sustained attention and sleep structure in the patient group. Previous work has shown that patients with MDD present abnormalities in sustained attention (Han et al. 2012; Rock et al. 2014) and polysomnography‐measured sleep structure (Riemann et al. 2020). The involvement of ACC function and corona radiata integrity in sustained attention has been documented (Sasson et al. 2012; Wu et al. 2017). It is generally accepted that the ACC is also a key brain region critically involved in both REM and non‐REM sleep (Maquet 2000), such that its function alteration might have an impact on sleep structure. Our mediation results indicate that higher serum CRP levels may lead to decreased ACC function and superior corona radiata integrity, which may in turn give rise to worse sustained attention in MDD patients; likewise, higher CRP may cause decreased ACC function that might alter non‐REM sleep (decreased N2% and increased N3%). In fact, circulating pro‐inflammatory cytokines may affect the brain through neural pathways involving the afferent vagus nerve, as well as humoral pathways crossing the blood–brain barrier (Lemogne et al. 2025). In the brain, pro‐inflammatory cytokine targets include both neurons and glial cells, particularly astrocytes and microglia, with the pro‐inflammatory effects leading to a reduced kynurenic acid‐to‐quinolinic acid ratio. These changes result in “sickness behaviors,” characterized by numerous symptoms including sleep and concentration disturbances (Lemogne et al. 2025), which overlap with those of MDD. In sum, these findings provide insights into the neuroanatomic substrates underlying the detrimental effects of circulating inflammation on sustained attention and sleep structure in MDD, which may hold value as important biomarkers for monitoring and/or predicting therapeutic responses of targeting inflammatory processes to improve these clinical manifestations.

### 4.1 Limitations

Our study has several limitations that must be considered. First, this is a cross‐sectional study, hampering the possibility of inferring causality between determinant and outcome. Longitudinal studies will be required to establish the direction of causality. Second, serum CRP levels could be affected by many confounding factors, such as stress and physical activity, which should be measured and controlled in future research. Third, our patients were receiving antidepressants and had different illness durations, which may influence our interpretation. Although the results were preserved after controlling for antidepressant types and illness duration, their effects cannot be eliminated completely. Further investigation in first‐episode, medication‐naive patients with MDD is encouraged to validate our findings. Finally, the neuroimaging measures were computed based on our multi‐modal MRI data, whereas the transcriptome, neurotransmitter, and behavioral domain atlases were derived from publicly available datasets. Our spatial correlation analyses ignored such variability across individuals, which should be captured and examined in the future.

## 5 Conclusion

In conclusion, our data provide first empirical evidence for the neural correlates of peripheral inflammation in MDD and their transcriptomic architecture, neurochemical basis, and behavioral relevance, as well as demonstrate the mediative role of the neural correlates in accounting for the inflammatory effects on the clinical presentation of MDD. These findings may not only confirm the role of inflammation in the neuropathology of MDD, but also inform a novel conceptualization of targeting inflammatory processes to treat this disorder.

## Conflicts of Interest

The authors declare no conflicts of interest.

## Supporting information


**Data S1:** hbm70371‐sup‐0001‐supinfo1.xlsx.

**Data S2:** hbm70371‐sup‐0002‐supinfo2.xlsx.

**Data S3:** hbm70371‐sup‐0003‐supinfo3.xlsx.

**Data S4:** hbm70371‐sup‐0004‐supinfo4.xlsx.

**Data S5:** hbm70371‐sup‐0005‐supinfo5.docx.

## Data Availability

The data that support the findings of this study are available from the corresponding author upon reasonable request.
