# Supplementary material for: Neural Correlates of Peripheral Inflammation in Major Depressive Disorder and Their Transcriptomic Architecture, Neurochemical Basis, and Behavioral Relevance
Source: Hum Brain Mapp. 2025 Sep 27;46(14):e70371. doi: 10.1002/hbm.70371 (PMC12476116; doi:10.1002/hbm.70371)
Supplement: Supplementary file 5 — Data S5: hbm70371‐sup‐0005‐supinfo5.docx. [file HBM-46-e70371-s004.docx]

**Supplementary Materials**

**Materials and methods**

**Participants**

MDD patients were enrolled from Affiliated Psychological Hospital of Anhui Medical University. Healthy controls (HC) were recruited from the local community via poster advertisements. A total of 244 participants with right-handedness were included, consisting of 122 MDD patients and 122 sex- and age-matched HC. Two well-trained clinical psychiatrists confirmed the diagnoses of depression using the MINI-International Neuropsychiatric Interview (M.I.N.I.) in accordance with the International Classification of Diseases (ICD-10) criteria (Sheehan et al., 1998). HC were carefully screened to confirm an absence of any psychiatric illness using the M.I.N.I. The exclusion criteria for all participants included 1) the presence of other psychiatric disorders such as substance-induced mood disorder, bipolar disorder, anxiety disorders, schizophrenia, substance abuse or dependence; 2) a history of significant physical or neurological diseases; 3) a history of head injury with consciousness loss; 4) contraindications for MRI such as pregnancy. Additional exclusion criterion for HC was a family history of serious neurological or psychiatric illnesses among their first-degree relatives. It is noteworthy that one participant’s blood sample was missing, and participants with serum CRP values greater than mean + 3 × standard deviation (SD) or smaller than mean - 3 × SD (i.e., outliers, n = 5) were excluded. This brought the final sample into 238 participants, comprising 120 MDD patients and 118 HC. The 24-item Hamilton Rating Scale for Depression (HAMD) (Williams, 1988) and the 14-item Hamilton Rating Scale for Anxiety (HAMA) (Thompson, 2015) were utilized to evaluate the severity of depression and anxiety symptoms. Subjective sleep quality was assessed by using Pittsburg Sleep Quality Index (PSQI). All MDD patients were receiving regular antidepressant medications, including selective serotonin reuptake inhibitors (SSRIs), serotonin-norepinephrine reuptake inhibitors (SNRIs), and noradrenergic and specific serotonergic antidepressants (NaSSA).

**Cognitive assessment**

The stimuli were 2-, 3-, or 4-digit numbers in separate conditions, which yielded separate scores reflecting increasing memory load on digit span. Participants were asked to monitor numbers on a computer screen and respond to any consecutive presentation of identical stimuli by key pressing as quickly as possible. Responses to target trials (pairs that were identical and required a response) and catch trials (pairs that were similar but not identical) were scored as true and false positive responses. The main outcome variable of interest was *d*' -a well-established discrimination sensitivity index incorporating both true and false positive responses. CPT-IP-2, -3, and -4 represented *d*' values corresponding to the number of digits.

**Image acquisition**

MRI data were acquired using a 3.0-Tesla MR system (Discovery MR750w, General Electric, Milwaukee, WI, USA) with a 24-channel head coil. During scanning, tight but comfortable foam and earplugs were used to minimize head movement and scanner noise. All participants were instructed to relax, keep their eyes closed but not fall asleep, think of nothing in particular, and move as little as possible. All participants underwent a high-resolution three-dimensional T1-weighted brain volume (BRAVO) sequence with the following parameters: repetition time (TR) = 8.5 ms; echo time (TE) = 3.2 ms; inversion time (TI) = 450 ms; flip angle (FA) = 12 °; field of view (FOV) = 256 mm × 256 mm; matrix size = 256 × 256; slice thickness = 1 mm, no gap; voxel size = 1 mm × 1 mm × 1 mm; 188 sagittal slices; and acquisition time = 296 s. Resting-state blood-oxygen-level-dependent (BOLD) fMRI data were acquired using a gradient-echo single-shot echo planar imaging (GRE-SS-EPI) sequence with the following parameters: TR = 2000 ms; TE = 30 ms; FA = 90 °; FOV = 220 mm × 220 mm; matrix size = 64 × 64; slice thickness = 3 mm, slice gap = 1 mm; 35 interleaved axial slices; 185 volumes; and acquisition time = 370 s. DTI data were acquired using a spin-echo single-shot echo planar imaging (SE-SS-EPI) sequence with the following parameters: TR = 10000 ms; TE = 74 ms; flip angle = 90 °; FOV = 256 mm × 256 mm; matrix = 128 × 128; slice thickness = 3 mm without gap; 50 axial slices; 64 diffusion gradient directions (b = 1000 s/mm^2^) plus five b = 0 reference images; and acquisition time = 700 s. Routine T2-weighted images were also collected to exclude any organic brain abnormality. None of the participants were excluded for visually inspected imaging artifacts.

**fMRI data processing**

Resting-state fMRI data were preprocessed using Statistical Parametric Mapping (SPM12, <http://www.fil.ion.ucl.ac.uk/spm>) and Data Processing & Analysis for Brain Imaging (DPABI, <http://rfmri.org/dpabi>) (Yan et al., 2016). The first 10 volumes for each subject were discarded, and the remaining volumes were corrected for the acquisition time delay between slices. Realignment was then performed to correct the motion between time points. Head motion parameters were computed by estimating the translation in each direction and the angular rotation on each axis for each volume. All data were within the defined motion thresholds (i.e., translational or rotational motion parameters less than 2.0 mm or 2.0 ^°^). We also calculated frame-wise displacement (FD), which indexes the volume-to-volume changes in head position. Several nuisance covariates (the estimated motion parameters based on the Friston-24 model, the linear drift, the white matter signal, the cerebrospinal fluid signal, and the spike volumes with FD > 0.5 mm) were regressed out from the data. In the normalization step, individual structural images were firstly co-registered with the mean functional image; the transformed structural images were then segmented and normalized to the Montreal Neurological Institute (MNI) space using a high-level nonlinear warping algorithm, i.e., the diffeomorphic anatomical registration through exponentiated Lie algebra technique (J. Ashburner, 2007). Finally, each functional volume was spatially normalized to the MNI space using the deformation parameters estimated during the above step and resampled into a 3 mm isotropic voxel. After spatial normalization, all datasets were smoothed with a 6 mm full-width at half-maximum (FWHM) Gaussian kernel.

**DTI data processing**

For DTI data, standard processing steps were performed by using the FMRIB Software Library (FSL, www.fmrib.ox.ac.uk/fsl). First, eddy current distortion and head motion were corrected by registering the diffusion-weighted images to the first b0 image through the affine transformations (S. M. Smith et al., 2004). Second, the data were skull-stripped by using the FMRIB Brain Extraction Tool (S.M Smith, 2002). Finally, diffusion parameters including fractional anisotropy (FA), axial diffusivity (AD), radial diffusivity (RD), and mean diffusivity (MD) were calculated by using the DTIFIT toolbox at each voxel.

**Brain gene expression data processing**

We first updated the probe-to-gene annotations based on the latest available information from the National Center for Biotechnology Information (NCBI) using the Re-Annotator package (Arloth et al., 2015). With intensity-based filtering, we excluded probes that did not exceed the background noise in at least 50% of samples across all donors. Since multiple probes were used to measure expression level of a single gene, we further used the RNA-seq data as a reference to select probes. After excluding genes that do not overlap between RNA-seq and microarray datasets, we calculated the correlations between microarray and RNA-seq expression measures for the remaining genes. After excluding probes with low correlations (*r* < 0.2), a representative probe was selected for a gene based on the highest correlation with the RNA-seq data. In this study, we only included the tissue samples in the left cerebral cortex. On the one hand, all six donors had expression data in the left hemisphere, but only two donors had samples in the right hemisphere. On the other hand, the inclusion of subcortical samples might introduce potential biases because of substantial gene expression divergence between cortical and subcortical regions (M. J. Hawrylycz et al., 2012). To account for potential between-sample differences and donor-specific effects in gene expression, we performed both within-sample cross-gene and within-gene cross-sample normalization by using the scaled robust sigmoid normalization method. Differential stability (DS) is a measure of consistent regional variation across donor brains. Prior research has suggested that genes with high DS scores exhibit more consistent spatial expression patterns between donors and are enriched for brain-related biological function (M. Hawrylycz et al., 2015). Because gene expression conservation across subjects is a prerequisite for the transcriptome-neuroimaging spatial correlations, we only selected genes with relatively more conserved expression patterns for analysis. To achieve this goal, we ranked the genes by their DS values and chose the 50% of the highest DS genes for analysis. After these processing steps, we obtained normalized expression data of 5013 genes for 1280 tissue samples, resulting in a sample × gene matrix of 1280 × 5013. Because the voxel-wise CRP-fALFF correlations were performed within a gray matter mask, we further restricted our analyses to the samples within this mask, resulting in a final sample × gene matrix of 871 × 5013.

We adopted transcriptome-neuroimaging spatial correlation and the newly developed ensemble-based GCEA to investigate the transcriptomic architecture underlying the neural correlates of CRP. Specifically, we drew a spherical region (radius = 3 mm) centered at the Montreal Neurological Institute (MNI) coordinate of a given brain tissue sample and extracted the average *t*-value of voxels within the sphere from the statistical *t* map for the correlations between CRP and fALFF. Then, cross-sample Pearson correlations between gene expression and *t*-values were performed in a gene-wise manner, yielding 5013 spatial correlation coefficients (henceforth referred to as gene scores). In accordance with the Fulcher et al. study (Fulcher et al., 2021), we conducted neuroimaging-spatial ensemble-based GCEA for these gene scores in the following way. First, updated Gene Ontology (GO) term hierarchy and annotation files were obtained from the GO (http://geneontology.org/) (M. Ashburner et al., 2000) on 11th July 2022. Second, direct gene-to-category annotations were performed for the 5013 AHBA genes, and we restricted our analyses to GO categories with 10-200 annotations. Third, the gene scores were agglomerated at the level of GO categories as a mean score of genes annotated to each GO category. Fourth, we adopted the brainSMASH toolbox (<https://github.com/murraylab/brainsmash>), based on the spatial-lag model (Burt et al., 2020), to generate 10000 surrogate maps with spatial autocorrelation matched to that of the *t* map. Fifth, spatial correlations between gene expression and the 10000 spatial autocorrelation-preserving surrogate maps were carried out to construct a null distribution (i.e., neuroimaging-spatial ensemble-based null model) of mean gene scores for each GO category. Finally, statistical significance of a GO category was assessed by comparing the GO category score derived from the real data to the neuroimaging-spatial ensemble-based null. The significance level was set at two-sided *P* < 0.05 (i.e., higher or lower than the null).

**Sensitivity analysis**

We assigned AHBA gene expression to the Human Brainnetome Atlas, resulting in 102 regions with available gene expression. Then, the spatial associations between gene expression and the *t*-values were examined using partial least squares (PLS) regression, a multivariate statistical technique that can predict a set of dependent variables from a set of independent variables (Abdi et al., 2013). PLS regression is technically well suited to the high collinearity of gene expression data and has been extensively applied to transcriptome-neuroimaging association research (Hansen et al., 2021; Romero-Garcia et al., 2020; Song et al., 2023; Thomas et al., 2021). In our analysis, the gene expression matrix was defined as the independent variables, and the *t*-value vector was designated as the dependent variables. PLS regression identified the first PLS component where the weighted sum of gene expression (gene scores) were most strongly correlated with the *t*-values. Pearson's correlation coefficient between the gene scores and the *t*-values was calculated, and its statistical significance was assessed using a spatial autocorrelation-preserving permutation testing (5000 permutations).

Gene contribution was estimated with gene loading, that is, Pearson's correlation coefficient between an individual gene's expression pattern and PLS-derived gene scores across regions. Then, genes were segregated based on the sign of their loadings, where positive genes refer to genes with positive loadings and negative genes refer to those with negative loadings. Strongly contributing genes were defined as those among the top 25% of the positive and negative genes, which were termed PLS+ and PLS- genes. To better understand the biological significance of PLS+ and PLS- genes, we conducted gene enrichment analysis, i.e., functional annotation. The functional annotation was performed with the ToppGene portal (<https://toppgene.cchmc.org/>) (Chen et al., 2009). Gene ontology (GO) was utilized to determine the biological functions including molecular functions (MFs), biological processes (BPs), and cellular components (CCs). Fisher's exact tests were used to evaluate their statistical significance. Multiple comparisons were adjusted by the Benjamini and Hochberg method for false discovery rate (FDR) with a corrected significance level of *P* < 0.05.**Table S1.** Demographic information of the six adult donors in the AHBA

| Donor | Age (years) | Sex | Ethnicity | Hemisphere | Post-mortem interval (h) |
| --- | --- | --- | --- | --- | --- |
| H0351.2001 | 24 | Male | African American | Both | 23 |
| H0351.2002 | 39 | Male | African American | Both | 10 |
| H0351.1009 | 57 | Male | Caucasian | Left | 25.5 |
| H0351.1012 | 31 | Male | Caucasian | Left | 17.5 |
| H0351.1015 | 49 | Female | Hispanic | Left | 30 |
| H0351.1016 | 55 | Male | Caucasian | Left | 18 |

Abbreviations: AHBA, Allen Human Brain Atlas. **Table S2.** Receptor/transporter maps

| Receptor/ transporter | Neurotransmitter | Tracer | Modality | Subjects | Source and reference |
| --- | --- | --- | --- | --- | --- |
| 5HT1a_1 | serotonin | (^11^C)WAY-100635 | PET | 35 | (Savli et al., 2012) |
| 5HT1b_1 | serotonin | (^11^C)P943 | PET | 23 |  |
| 5HT2a_1 | serotonin | (^18^F)altanserin | PET | 19 |  |
| SERT_1 | serotonin | (^11^C)DASB | PET | 18 |  |
| SERT_2 | serotonin | (^11^C)MADAM | PET | 10 | https://www.nitrc.org/projects/ki-5htt |
| SERT_3 | serotonin | (^11^C)DASB | PET | 100 | (Beliveau et al., 2017) |
| 5HT1a_2 | serotonin | (^11^C)CUMI-101 | PET | 8 |  |
| 5HT1b_2 | serotonin | (^11^C)AZ10419369 | PET | 36 |  |
| 5HT2a_2 | serotonin | (^11^C)Cimbi-36 | PET | 29 |  |
| 5HT4 | serotonin | (^11^C)SB207145 | PET | 59 |  |
| mGluR5_1 | glutamate | (^11^C)ABP688 | PET | 22 | PI: Pedro Rosa-Neto |
| mGluR5_2 | glutamate | (^11^C)ABP688 | PET | 28 | (DuBois et al., 2016) |
| mGluR5_3 | glutamate | (^11^C)ABP688 | PET | 73 | (Smart et al., 2019) |
| VAChT_1 | acetylcholine | (^18^ F) FEOBV | PET | 18 | (Aghourian et al., 2017) |
| VAChT_2 | acetylcholine | (^18^F)FEOBV | PET | 4 | PI: Lauri Tuominen & Synthia Guimond |
| VAChT_3 | acetylcholine | (^18^F)FEOBV | PET | 5 | (Bedard et al., 2019) |
| MOR_1 | opioid | (^11^C)carfentanil | PET | 204 | (Kantonen et al., 2020) |
| MOR_2 | opioid | (^11^C)carfentanil | PET | 39 | (Turtonen et al., 2021) |
| CB1 | cannabinoid | (^11^C)OMAR | PET | 77 | (Normandin et al., 2015) |
| D1 | dopamine | (^11^C)SCH23390 | PET | 13 | (Kaller et al., 2017) |
| D2_1 | dopamine | (^11^C)Raclopride | PET | 7 | (Alakurtti et al., 2015) |
| D2_2 | dopamine | (^11^C)FLB-457 | PET | 49 | (Zakiniaeiz et al., 2019) |
| NAT | noradrenaline | (^11^C)OMRB | PET | 20 | (Hesse et al., 2017) |
| DAT | dopamine | (^123^I)FP-CIT | SPECT | 174 | (Dukart et al., 2018) |
| GABAa_1 | GABA | (^11^C)flumazenil | PET | 6 |  |
| GABAa_2 | GABA | (^11^C)flumazenil | PET | 16 | (Norgaard et al., 2021) |
| FDOPA | fluorodopa | (^123^I)Ioflupane | SPECT | 26 | (Garcia-Gomez et al., 2013) |

Abbreviations: 5HT, 5-hydroxytryptamine; SERT, serotonin transporter; mGluR, metabotropic glutamate receptor; VAChT, vesicular acetylcholine transporter; MOR, mu opioid receptor; CB, cannabinoid; D, dopamine; NAT, noradrenaline transporter; DAT, dopamine transporter; GABAa, gamma-aminobutyric acid a; FDOPA, Fluorodopa; PET, positron emission tomography; SPECT, single photon emission computed tomography.**Table S3.** Correlations between CRP and clinical variables in the patient and control groups, respectively

|  | CRP (ml/L) | |
| --- | --- | --- |
|  | *pr* | *P* |
| Patient group |  |  |
| HAMD | -0.069 | 0.462 |
| HAMA | -0.118 | 0.204 |
| CPT-IP-2 | -0.038 | 0.683 |
| CPT-IP-3 | -0.125 | 0.178 |
| CPT-IP-4 | -0.026 | 0.777 |
| PSQI^#^ | -0.135 | 0.151 |
| Sleep efficiency^*^ | -0.022 | 0.819 |
| N1%^*^ | -0.071 | 0.449 |
| N2%^*^ | -0.017 | 0.853 |
| N3%^*^ | 0.044 | 0.639 |
| REM%^*^ | 0.094 | 0.318 |
| Control group |  |  |
| CPT-IP-2 | -0.161 | 0.086 |
| CPT-IP-3 | 0.032 | 0.736 |
| CPT-IP-4 | -0.025 | 0.791 |

Abbreviations: CRP, C-reactive protein; HAMD, Hamilton Rating Scale for Depression; HAMA, Hamilton Rating Scale for Anxiety; CPT-IP, Continuous Performance Task-Identical Pairs; PSQI, Pittsburgh Sleep Quality Index; REM, rapid eye movement; *pr*, partial correlation coefficient.

^#^ The data are available for 119 from 120 patients.

^*^ The data are available for 118 from 120 patients.

**Table S4.** Group differences in the correlations between C-reactive protein and neuroimaging measures

| Neuroimaging measures | MDD | HC | *Z* | *P* |
| --- | --- | --- | --- | --- |
|  | *pr* | *pr* |  |  |
| fALFF of R-PreCG | 0.405 | -0.032 | 3.520 | < 0.001 |
| fALFF of ACC | -0.384 | -0.166 | -1.810 | 0.073 |
| fALFF of MCC | -0.391 | -0.141 | -2.060 | 0.039 |
| AD of L-SS | -0.317 | -0.044 | -2.160 | 0.031 |
| AD of L-PLIC | -0.190 | -0.024 | -1.280 | 0.200 |
| AD of L-RLIC | -0.285 | -0.074 | -1.660 | 0.097 |
| AD of L-PTR | -0.239 | -0.073 | -1.300 | 0.194 |
| AD of R-PTR | -0.213 | 0.022 | -1.810 | 0.073 |
| AD of BCC | -0.363 | -0.092 | -2.190 | 0.029 |
| AD of SCC | -0.377 | -0.062 | -2.540 | 0.011 |
| AD of L-SCR | -0.230 | -0.120 | -0.860 | 0.390 |
| AD of R-SCR | -0.239 | 0.104 | -2.650 | 0.008 |
| AD of L-PCR | -0.252 | 0.080 | -2.570 | 0.010 |
| AD of R-PCR | -0.275 | 0.161 | -3.380 | < 0.001 |
| AD of R-SLF | -0.215 | 0.016 | -1.780 | 0.075 |

Abbreviations: MDD, major depressive disorder; fALFF, fractional amplitude of low frequency fluctuations; AD, axial diffusivity; PreCG, precentral gyrus; ACC, anterior cingulate cortex; MCC, middle cingulate cortex; SS, sagittal stratum; PLIC, posterior limb of internal capsule; RLIC, retrolenticular part of internal capsule; PTR, posterior thalamic radiation; BCC, body of corpus callosum; SCC, splenium of corpus callosum; SCR, superior corona radiate; PCR, posterior corona radiate; SLF, superior longitudinal fasciculus; L, left; R, right; *pr*, partial correlation coefficient.

**Table S5.** Correlations between neuroimaging measures and symptom as well as cognition in patients with major depressive disorder

| Neuroimaging measures | HAMD | | HAMA | | CPT-IP-2 | | CPT-IP-3 | | CPT-IP-4 | |
| --- | --- | --- | --- | --- | --- | --- | --- | --- | --- | --- |
|  | *pr* | *P* | *pr* | *P* | *pr* | *P* | *pr* | *P* | *pr* | *P* |
| fALFF of R-PreCG | -0.073 | 0.436 | -0.107 | 0.253 | -0.092 | 0.327 | -0.021 | 0.822 | -0.007 | 0.944 |
| fALFF of B-ACC | -0.035 | 0.705 | 0.014 | 0.882 | 0.225 | 0.015 | 0.217 | 0.019 | 0.002 | 0.979 |
| fALFF of B-MCC | -0.128 | 0.170 | -0.045 | 0.635 | 0.140 | 0.135 | 0.044 | 0.636 | 0.061 | 0.513 |
| AD of L-SS | 0.021 | 0.823 | -0.048 | 0.611 | 0.024 | 0.796 | -0.047 | 0.615 | 0.206 | 0.026 |
| AD of L-PLIC | -0.075 | 0.421 | -0.135 | 0.148 | -0.036 | 0.699 | 0.070 | 0.454 | 0.137 | 0.141 |
| AD of L-RLIC | -0.044 | 0.641 | -0.006 | 0.946 | 0.079 | 0.400 | 0.040 | 0.669 | 0.122 | 0.192 |
| AD of L-PTR | -0.049 | 0.597 | -0.003 | 0.972 | 0.120 | 0.198 | 0.055 | 0.558 | 0.140 | 0.133 |
| AD of R-PTR | 0.016 | 0.861 | -0.042 | 0.656 | 0.056 | 0.546 | 0.012 | 0.894 | 0.203 | 0.028 |
| AD of BCC | -0.002 | 0.986 | -0.009 | 0.922 | 0.152 | 0.103 | 0.162 | 0.081 | 0.155 | 0.096 |
| AD of SCC | 0.061 | 0.515 | 0.105 | 0.262 | 0.159 | 0.086 | 0.147 | 0.113 | 0.151 | 0.105 |
| AD of L-SCR | 0.005 | 0.959 | -0.041 | 0.662 | 0.152 | 0.102 | 0.073 | 0.432 | 0.228 | 0.013 |
| AD of R-SCR | -0.058 | 0.537 | -0.002 | 0.980 | 0.037 | 0.691 | 0.060 | 0.520 | 0.122 | 0.189 |
| AD of L-PCR | -0.056 | 0.547 | -0.051 | 0.582 | -0.033 | 0.721 | -0.044 | 0.635 | 0.037 | 0.692 |
| AD of R-PCR | -0.161 | 0.082 | -0.040 | 0.667 | -0.074 | 0.428 | -0.083 | 0.372 | 0.045 | 0.633 |
| AD of R-SLF | 0.029 | 0.754 | -0.033 | 0.720 | 0.113 | 0.224 | 0.042 | 0.651 | 0.206 | 0.026 |

Abbreviations: HAMD, Hamilton Rating Scale for Depression; HAMA, Hamilton Rating Scale for Anxiety; CPT-IP, Continuous Performance Task-Identical Pairs; fALFF, fractional amplitude of low-frequency fluctuations; AD, axial diffusivity; PreCG, precentral gyrus; ACC, anterior cingulate cortex; MCC, middle cingulate cortex; SS, sagittal stratum; PLIC, posterior limb of internal capsule; RLIC, retrolenticular part of internal capsule; PTR, posterior thalamic radiation; BCC, body of corpus callosum; SCC, splenium of corpus callosum; SCR, superior corona radiate; PCR, posterior corona radiate; SLF, superior longitudinal fasciculus; B, bilateral; L, left; R, right; *pr*, partial correlation coefficient.**Table S6.** Correlations between neuroimaging measures and sleep in patients with major depressive disorder

| Neuroimaging measures | PSQI^#^ | | Sleep efficiency^*^ | | N1%^*^ | | N2%^*^ | | N3%^*^ | | REM%^*^ | |
| --- | --- | --- | --- | --- | --- | --- | --- | --- | --- | --- | --- | --- |
|  | *pr* | *P* | *pr* | *P* | *pr* | *P* | *pr* | *P* | *pr* | *P* | *pr* | *P* |
| fALFF of R-PreCG | -0.038 | 0.691 | 0.135 | 0.152 | 0.027 | 0.775 | 0.045 | 0.632 | -0.099 | 0.294 | -0.021 | 0.828 |
| fALFF of B-ACC | 0.062 | 0.509 | -0.038 | 0.688 | -0.048 | 0.613 | 0.247 | 0.008 | -0.338 | < 0.001 | -0.050 | 0.601 |
| fALFF of B-MCC | 0.085 | 0.368 | -0.078 | 0.407 | 0.013 | 0.892 | 0.041 | 0.667 | -0.092 | 0.333 | 0.001 | 0.992 |
| AD of L-SS | -0.152 | 0.105 | 0.032 | 0.730 | 0.073 | 0.438 | 0.006 | 0.947 | -0.080 | 0.398 | -0.033 | 0.724 |
| AD of L-PLIC | -0.170 | 0.069 | -0.141 | 0.134 | 0.092 | 0.327 | -0.138 | 0.141 | 0.084 | 0.372 | 0.048 | 0.612 |
| AD of L-RLIC | -0.062 | 0.512 | -0.196 | 0.036 | 0.120 | 0.200 | -0.033 | 0.729 | 0.010 | 0.912 | -0.127 | 0.175 |
| AD of L-PTR | -0.004 | 0.965 | -0.078 | 0.409 | 0.142 | 0.129 | -0.012 | 0.899 | -0.071 | 0.448 | -0.110 | 0.243 |
| AD of R-PTR | 0.076 | 0.419 | 0.042 | 0.653 | 0.062 | 0.512 | 0.026 | 0.780 | -0.017 | 0.859 | -0.129 | 0.171 |
| AD of BCC | -0.009 | 0.927 | -0.058 | 0.535 | 0.056 | 0.553 | -0.017 | 0.854 | -0.092 | 0.327 | 0.056 | 0.550 |
| AD of SCC | -0.019 | 0.839 | -0.024 | 0.800 | 0.096 | 0.310 | 0.059 | 0.534 | -0.115 | 0.221 | -0.135 | 0.149 |
| AD of L-SCR | -0.100 | 0.287 | -0.039 | 0.675 | 0.017 | 0.856 | -0.111 | 0.237 | 0.112 | 0.233 | 0.075 | 0.427 |
| AD of R-SCR | 0.014 | 0.882 | -0.053 | 0.574 | 0.038 | 0.683 | -0.014 | 0.884 | -0.014 | 0.880 | -0.014 | 0.880 |
| AD of L-PCR | 0.024 | 0.797 | 0.027 | 0.773 | -0.054 | 0.563 | -0.012 | 0.895 | 0.067 | 0.478 | 0.032 | 0.736 |
| AD of R-PCR | 0.131 | 0.164 | -0.014 | 0.878 | 0.002 | 0.985 | 0.043 | 0.651 | 0.014 | 0.878 | -0.108 | 0.252 |
| AD of R-SLF | -0.110 | 0.243 | 0.058 | 0.536 | 0.093 | 0.324 | -0.028 | 0.765 | 0.010 | 0.915 | -0.095 | 0.313 |

Abbreviations: PSQI, Pittsburgh Sleep Quality Index; REM, rapid eye movement; fALFF, fractional amplitude of low-frequency fluctuations; AD, axial diffusivity; PreCG, precentral gyrus; ACC, anterior cingulate cortex; MCC, middle cingulate cortex; SS, sagittal stratum; PLIC, posterior limb of internal capsule; RLIC, retrolenticular part of internal capsule; PTR, posterior thalamic radiation; BCC, body of corpus callosum; SCC, splenium of corpus callosum; SCR, superior corona radiate; PCR, posterior corona radiate; SLF, superior longitudinal fasciculus; B, bilateral; L, left; R, right; *pr*, partial correlation coefficient.

^#^ The data are available for 119 from 120 patients.

^*^ The data are available for 118 from 120 patients.

**Table S7.** Correlations between CRP and neuroimaging measures in patients with major depressive disorder after additionally adjusting for BMI

| Neuroimaging measures | CRP (ml/L) | |
| --- | --- | --- |
|  | *pr* | *P* |
| fALFF of R-PreCG | 0.383 | < 0.001 |
| fALFF of B-ACC | -0.313 | < 0.001 |
| fALFF of B-MCC | -0.365 | < 0.001 |
| AD of L-SS | -0.288 | 0.002 |
| AD of L-PLIC | -0.228 | 0.014 |
| AD of L-RLIC | -0.294 | 0.001 |
| AD of L-PTR | -0.206 | 0.027 |
| AD of R-PTR | -0.206 | 0.027 |
| AD of BCC | -0.303 | < 0.001 |
| AD of SCC | -0.280 | 0.002 |
| AD of L-SCR | -0.262 | 0.005 |
| AD of R-SCR | -0.268 | 0.004 |
| AD of L-PCR | -0.288 | 0.002 |
| AD of R-PCR | -0.307 | < 0.001 |
| AD of R-SLF | -0.235 | 0.011 |

Abbreviations: CRP, C-reactive protein; BMI, body mass index; fALFF, fractional amplitude of low-frequency fluctuations; AD, axial diffusivity; PreCG, precentral gyrus; ACC, anterior cingulate cortex; MCC, middle cingulate cortex; SS, sagittal stratum; PLIC, posterior limb of internal capsule; RLIC, retrolenticular part of internal capsule; PTR, posterior thalamic radiation; BCC, body of corpus callosum; SCC, splenium of corpus callosum; SCR, superior corona radiate; PCR, posterior corona radiate; SLF, superior longitudinal fasciculus; B, bilateral; L, left; R, right; *pr*, partial correlation coefficient.

**Table S8.** Correlations between neuroimaging measures and clinical variables in patients with major depressive disorder after additionally adjusting for BMI

|  | fALFF of B-ACC | | AD of L-SCR | |
| --- | --- | --- | --- | --- |
|  | *pr* | *P* | *pr* | *P* |
| CPT-IP-2 | 0.207 | 0.026 | - | - |
| CPT-IP-3 | 0.189 | 0.043 | - | - |
| CPT-IP-4 | - | - | 0.230 | 0.013 |
| N2%^*^ | 0.228 | 0.015 | - | - |
| N3%^*^ | -0.348 | < 0.001 | - | - |

Abbreviations: BMI, body mass index; CPT-IP, Continuous Performance Task-Identical Pairs; fALFF, fractional amplitude of low-frequency fluctuations; AD, axial diffusivity; ACC, anterior cingulate cortex; SCR, superior corona radiate; B, bilateral; L, left; *pr*, partial correlation coefficient.

^*^ The data are available for 118 from 120 patients.

**Table S9.** Mediation analyses with CRP, neuroimaging measures and clinical variables as independent, mediating and dependent variables in patients with major depressive disorder after additionally adjusting for BMI

| Independent variable | Mediating variable | Dependent variable | Indirect effect | Standard error | 95% confidence interval |
| --- | --- | --- | --- | --- | --- |
| CRP | fALFF of B-ACC | CPT-IP-2 | -0.0524 | 0.0240 | -0.1097, -0.0131 |
| CRP | fALFF of B-ACC | CPT-IP-3 | -0.0402 | 0.0225 | -0.0918, -0.0021 |
| CRP | AD of L-SCR | CPT-IP-4 | -0.0369 | 0.0231 | -0.0966, -0.0062 |
| CRP | fALFF of B-ACC | N2%^*^ | -0.9012 | 0.4056 | -1.9337, -0.2624 |
| CRP | fALFF of B-ACC | N3%^*^ | 0.7074 | 0.2562 | 0.3003, 1.3379 |

Abbreviations: CRP, C-reactive protein; BMI, body mass index; CPT-IP, Continuous Performance Task-Identical Pairs; fALFF, fractional amplitude of low-frequency fluctuations; AD, axial diffusivity; ACC, anterior cingulate cortex; SCR, superior corona radiate; B, bilateral; L, left.

^*^ The data are available for 118 from 120 patients.

**Table S10.** Correlations between CRP and neuroimaging measures in patients with major depressive disorder after additionally controlling for antidepressant types and illness duration

| Neuroimaging measures | CRP (ml/L) | |
| --- | --- | --- |
|  | *pr* | *P* |
| fALFF of R-PreCG | 0.410 | < 0.001 |
| fALFF of B-ACC | -0.388 | < 0.001 |
| fALFF of B-MCC | -0.392 | < 0.001 |
| AD of L-SS | -0.315 | < 0.001 |
| AD of L-PLIC | -0.187 | 0.045 |
| AD of L-RLIC | -0.287 | 0.002 |
| AD of L-PTR | -0.237 | 0.011 |
| AD of R-PTR | -0.212 | 0.023 |
| AD of BCC | -0.361 | < 0.001 |
| AD of SCC | -0.380 | < 0.001 |
| AD of L-SCR | -0.228 | 0.014 |
| AD of R-SCR | -0.235 | 0.011 |
| AD of L-PCR | -0.247 | 0.008 |
| AD of R-PCR | -0.273 | 0.003 |
| AD of R-SLF | -0.212 | 0.023 |

Abbreviations: CRP, C-reactive protein; fALFF, fractional amplitude of low-frequency fluctuations; AD, axial diffusivity; PreCG, precentral gyrus; ACC, anterior cingulate cortex; MCC, middle cingulate cortex; SS, sagittal stratum; PLIC, posterior limb of internal capsule; RLIC, retrolenticular part of internal capsule; PTR, posterior thalamic radiation; BCC, body of corpus callosum; SCC, splenium of corpus callosum; SCR, superior corona radiate; PCR, posterior corona radiate; SLF, superior longitudinal fasciculus; B, bilateral; L, left; R, right; *pr*, partial correlation coefficient.**Table S11.** Correlations between neuroimaging measures and clinical variables in patients with major depressive disorder after additionally controlling for antidepressant types and illness duration

|  | fALFF of B-ACC | | AD of L-SCR | |
| --- | --- | --- | --- | --- |
|  | *pr* | *P* | *pr* | *P* |
| CPT-IP-2 | 0.214 | 0.022 |  |  |
| CPT-IP-3 | 0.218 | 0.020 |  |  |
| CPT-IP-4 |  |  | 0.233 | 0.012 |
| N2%^*^ | 0.249 | 0.008 |  |  |
| N3%^*^ | -0.355 | < 0.001 |  |  |

Abbreviations: CPT-IP, Continuous Performance Task-Identical Pairs; fALFF, fractional amplitude of low-frequency fluctuations; AD, axial diffusivity; ACC, anterior cingulate cortex; SCR, superior corona radiate; B, bilateral; L, left; *pr*, partial correlation coefficient.

^*^ The data are available for 118 from 120 patients.**Table S12.** Mediation analyses with CRP, neuroimaging measures and clinical variables as independent, mediating and dependent variables in patients with major depressive disorder after additionally controlling for antidepressant types and illness duration

| Independent variable | Mediating variable | Dependent variable | Indirect effect | Standard error | 95% confidence interval |
| --- | --- | --- | --- | --- | --- |
| CRP | fALFF of B-ACC | CPT-IP-2 | -0.0588 | 0.0282 | -0.1243, -0.0117 |
| CRP | fALFF of B-ACC | CPT-IP-3 | -0.0486 | 0.0253 | -0.1042, -0.0037 |
| CRP | AD of L-SCR | CPT-IP-4 | -0.0275 | 0.0204 | -0.0868, -0.0037 |
| CRP | fALFF of B-ACC | N2%^*^ | -1.1007 | 0.4396 | -2.1453, -0.3880 |
| CRP | fALFF of B-ACC | N3%^*^ | 0.8437 | 0.2565 | 0.4177, 1.4743 |

Abbreviations: CRP, C-reactive protein; CPT-IP, Continuous Performance Task-Identical Pairs; fALFF, fractional amplitude of low-frequency fluctuations; AD, axial diffusivity; ACC, anterior cingulate cortex; SCR, superior corona radiate; B, bilateral; L, left.

^*^ The data are available for 118 from 120 patients.


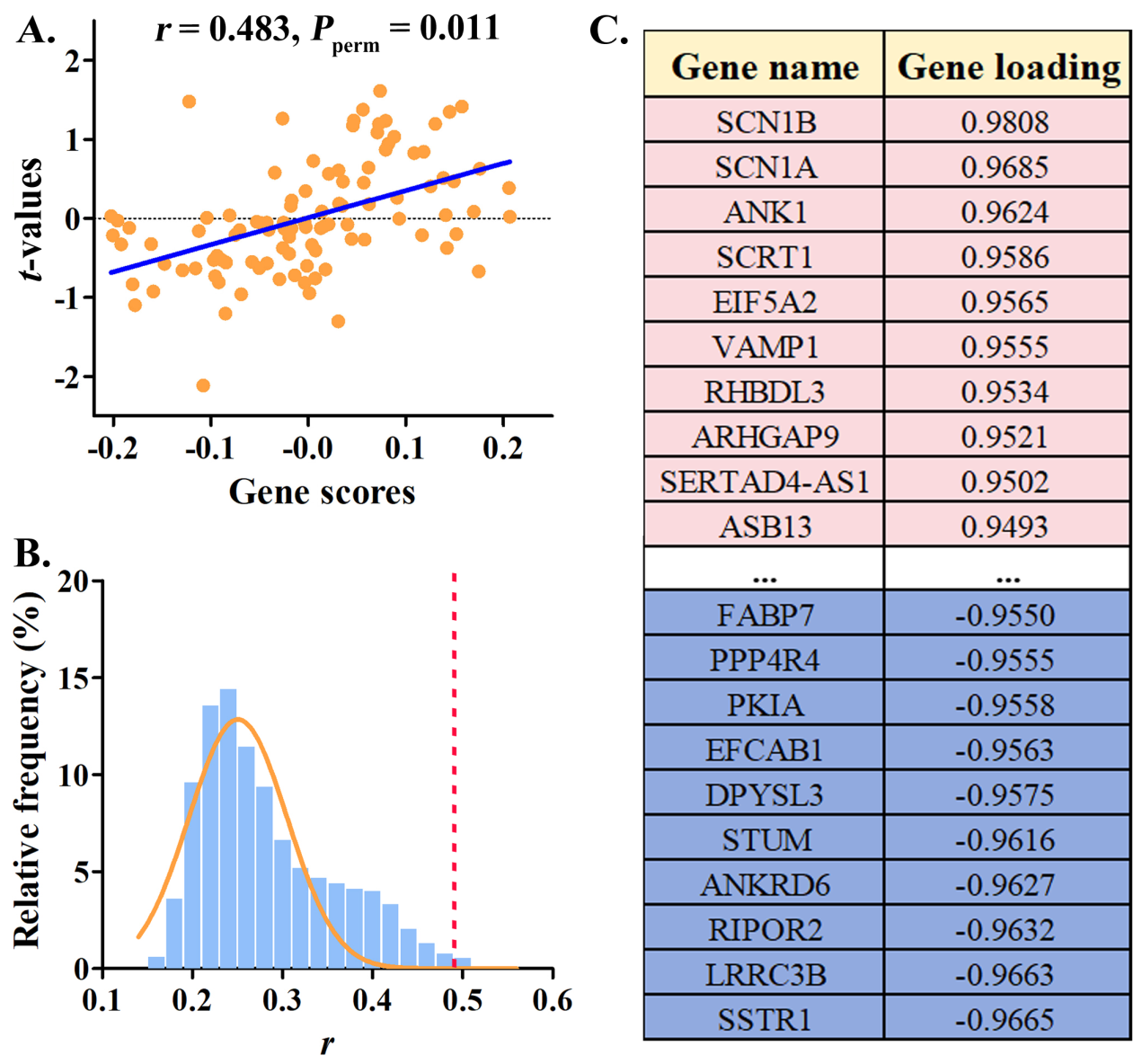


**Figure S1.** Gene expression and the neural correlates of CRP. (A) Scatterplot of the gene scores (identified by PLS regression) versus the *t*-values with each point representing a region with available gene expression. (B) Histogram of permutation distribution showing that the correlation (dotted line) between the gene scores and the *t*-values was significantly greater than expected by chance. (C) Illustrative example of the loadings (gene contributions) assigned to representative genes. Genes with the highest positive loadings are colored in pink and those with the lowest negative loadings are colored in blue. Abbreviations: CRP, C-reactive protein; PLS, partial least squares.


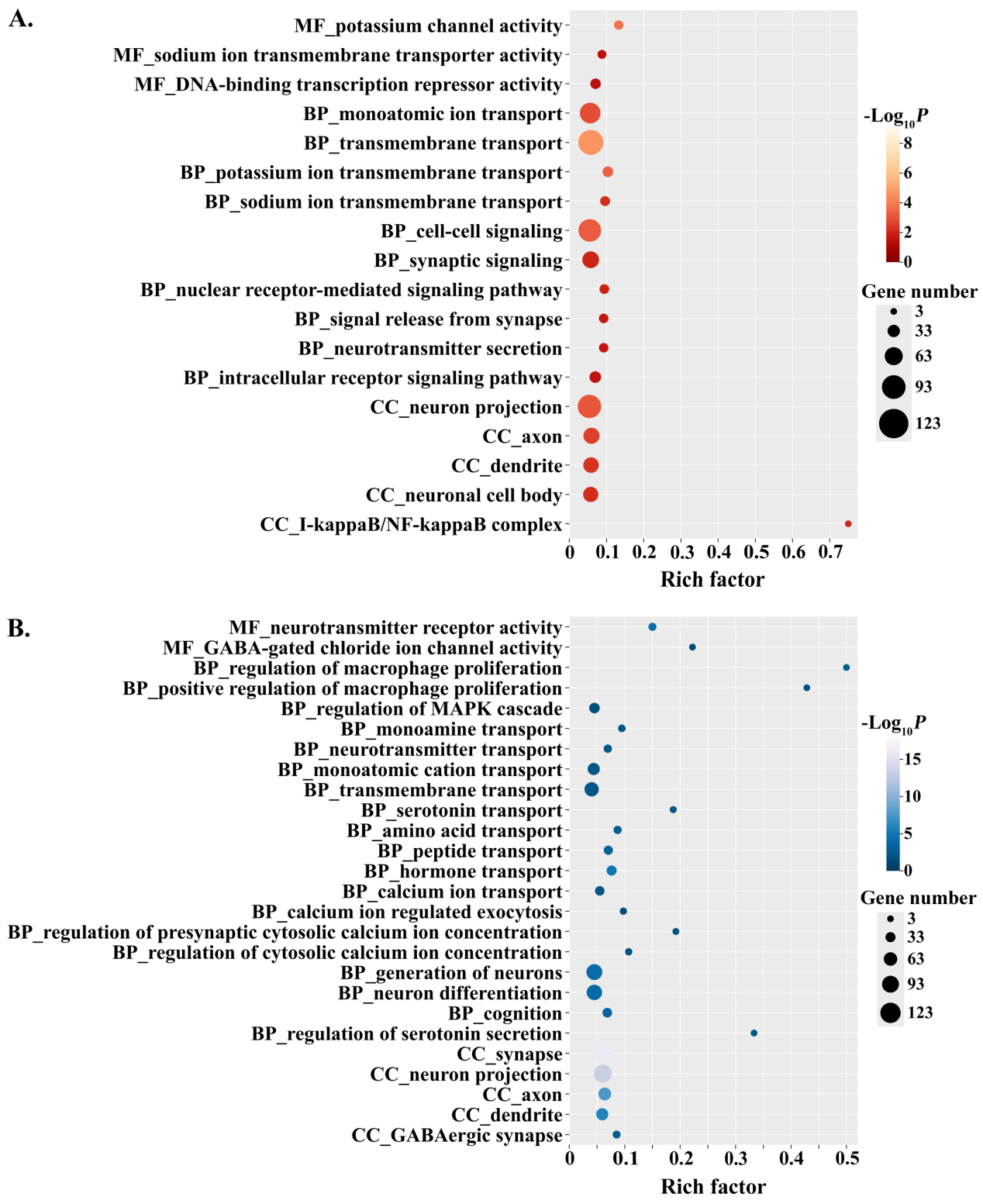


**Figure S2.** Enrichment analyses of the strongly contributing genes. Functional annotation of 698 PLS+ genes (the top 25% of genes with positive loadings) (A) and 555 PLS- genes (the top 25% of genes with negative loadings) (B). For each bubble chart, the x-axis denotes the rich factor and the y-axis denotes items from the GO. The rich factor refers to the ratio of the number of PLS+ or PLS- genes annotated to the item to the number of all genes annotated to the item. The bubble size represents the number of genes overlapping with those belonging to each item, and the bubble color represents the -Log_10_ *P* with the *P* value corrected by the FDR-BH method. Abbreviations: GO, gene ontology; MF, molecular function; BP, biological process; CC, cellular component; FDR-BH, the Benjamini and Hochberg method for false discovery rate.**References**

Abdi, H., & Williams, L. J. (2013). Partial least squares methods: partial least squares correlation and partial least square regression. *Methods Mol Biol, 930*, 549-579. doi:10.1007/978-1-62703-059-5_23

Aghourian, M., Legault-Denis, C., Soucy, J. P., Rosa-Neto, P., Gauthier, S., Kostikov, A., . . . Bedard, M. A. (2017). Quantification of brain cholinergic denervation in Alzheimer's disease using PET imaging with [(18)F]-FEOBV. *Mol Psychiatry, 22*(11), 1531-1538. doi:10.1038/mp.2017.183

Alakurtti, K., Johansson, J. J., Joutsa, J., Laine, M., Backman, L., Nyberg, L., & Rinne, J. O. (2015). Long-term test-retest reliability of striatal and extrastriatal dopamine D2/3 receptor binding: study with [(11)C]raclopride and high-resolution PET. *J Cereb Blood Flow Metab, 35*(7), 1199-1205. doi:10.1038/jcbfm.2015.53

Arloth, J., Bader, D. M., Roh, S., & Altmann, A. (2015). Re-Annotator: Annotation Pipeline for Microarray Probe Sequences. *PLoS One, 10*(10), e0139516. doi:10.1371/journal.pone.0139516

Ashburner, J. (2007). A fast diffeomorphic image registration algorithm. *Neuroimage, 38*(1), 95-113. doi:10.1016/j.neuroimage.2007.07.007

Ashburner, M., Ball, C. A., Blake, J. A., Botstein, D., Butler, H., Cherry, J. M., . . . Sherlock, G. (2000). Gene ontology: tool for the unification of biology. The Gene Ontology Consortium. *Nat Genet, 25*(1), 25-29. doi:10.1038/75556

Bedard, M. A., Aghourian, M., Legault-Denis, C., Postuma, R. B., Soucy, J. P., Gagnon, J. F., . . . Montplaisir, J. (2019). Brain cholinergic alterations in idiopathic REM sleep behaviour disorder: a PET imaging study with (18)F-FEOBV. *Sleep Med, 58*, 35-41. doi:10.1016/j.sleep.2018.12.020

Beliveau, V., Ganz, M., Feng, L., Ozenne, B., Hojgaard, L., Fisher, P. M., . . . Knudsen, G. M. (2017). A High-Resolution In Vivo Atlas of the Human Brain’s Serotonin System. *J Neurosci, 37*(1), 120-128. doi:10.1523/JNEUROSCI.2830-16.2016

Burt, J. B., Helmer, M., Shinn, M., Anticevic, A., & Murray, J. D. (2020). Generative modeling of brain maps with spatial autocorrelation. *Neuroimage, 220*, 117038. doi:10.1016/j.neuroimage.2020.117038

Chen, J., Bardes, E. E., Aronow, B. J., & Jegga, A. G. (2009). ToppGene Suite for gene list enrichment analysis and candidate gene prioritization. *Nucleic Acids Res, 37*(Web Server issue), W305-311. doi:10.1093/nar/gkp427

DuBois, J. M., Rousset, O. G., Rowley, J., Porras-Betancourt, M., Reader, A. J., Labbe, A., . . . Kobayashi, E. (2016). Characterization of age/sex and the regional distribution of mGluR5 availability in the healthy human brain measured by high-resolution [(11)C]ABP688 PET. *Eur J Nucl Med Mol Imaging, 43*(1), 152-162. doi:10.1007/s00259-015-3167-6

Dukart, J., Holiga, S., Chatham, C., Hawkins, P., Forsyth, A., McMillan, R., . . . Sambataro, F. (2018). Cerebral blood flow predicts differential neurotransmitter activity. *Sci Rep, 8*(1), 4074. doi:10.1038/s41598-018-22444-0

Fulcher, B. D., Arnatkeviciute, A., & Fornito, A. (2021). Overcoming false-positive gene-category enrichment in the analysis of spatially resolved transcriptomic brain atlas data. *Nat Commun, 12*(1), 2669. doi:10.1038/s41467-021-22862-1

Garcia-Gomez, F. J., Garcia-Solis, D., Luis-Simon, F. J., Marin-Oyaga, V. A., Carrillo, F., Mir, P., & Vazquez-Albertino, R. J. (2013). [Elaboration of the SPM template for the standardization of SPECT images with 123I-Ioflupane]. *Rev Esp Med Nucl Imagen Mol, 32*(6), 350-356. doi:10.1016/j.remn.2013.02.009

Hansen, J. Y., Markello, R. D., Vogel, J. W., Seidlitz, J., Bzdok, D., & Misic, B. (2021). Mapping gene transcription and neurocognition across human neocortex. *Nat Hum Behav, 5*(9), 1240-1250. doi:10.1038/s41562-021-01082-z

Hawrylycz, M., Miller, J. A., Menon, V., Feng, D., Dolbeare, T., Guillozet-Bongaarts, A. L., . . . Lein, E. (2015). Canonical genetic signatures of the adult human brain. *Nat Neurosci, 18*(12), 1832-1844. doi:10.1038/nn.4171

Hawrylycz, M. J., Lein, E. S., Guillozet-Bongaarts, A. L., Shen, E. H., Ng, L., Miller, J. A., . . . Jones, A. R. (2012). An anatomically comprehensive atlas of the adult human brain transcriptome. *Nature, 489*(7416), 391-399. doi:10.1038/nature11405

Hesse, S., Becker, G. A., Rullmann, M., Bresch, A., Luthardt, J., Hankir, M. K., . . . Sabri, O. (2017). Central noradrenaline transporter availability in highly obese, non-depressed individuals. *Eur J Nucl Med Mol Imaging, 44*(6), 1056-1064. doi:10.1007/s00259-016-3590-3

Kaller, S., Rullmann, M., Patt, M., Becker, G. A., Luthardt, J., Girbardt, J., . . . Sabri, O. (2017). Test-retest measurements of dopamine D1-type receptors using simultaneous PET/MRI imaging. *Eur J Nucl Med Mol Imaging, 44*(6), 1025-1032. doi:10.1007/s00259-017-3645-0

Kantonen, T., Karjalainen, T., Isojarvi, J., Nuutila, P., Tuisku, J., Rinne, J., . . . Nummenmaa, L. (2020). Interindividual variability and lateralization of mu-opioid receptors in the human brain. *Neuroimage, 217*, 116922. doi:10.1016/j.neuroimage.2020.116922

Norgaard, M., Beliveau, V., Ganz, M., Svarer, C., Pinborg, L. H., Keller, S. H., . . . Knudsen, G. M. (2021). A high-resolution in vivo atlas of the human brain's benzodiazepine binding site of GABAA receptors. *Neuroimage, 232*, 117878. doi:10.1016/j.neuroimage.2021.117878

Normandin, M. D., Zheng, M. Q., Lin, K. S., Mason, N. S., Lin, S. F., Ropchan, J., . . . Huang, Y. (2015). Imaging the cannabinoid CB1 receptor in humans with [11C]OMAR: assessment of kinetic analysis methods, test-retest reproducibility, and gender differences. *J Cereb Blood Flow Metab, 35*(8), 1313-1322. doi:10.1038/jcbfm.2015.46

Romero-Garcia, R., Seidlitz, J., Whitaker, K. J., Morgan, S. E., Fonagy, P., Dolan, R. J., . . . Bullmore, E. T. (2020). Schizotypy-Related Magnetization of Cortex in Healthy Adolescence Is Colocated With Expression of Schizophrenia-Related Genes. *Biol Psychiatry, 88*(3), 248-259. doi:10.1016/j.biopsych.2019.12.005

Savli, M., Bauer, A., Mitterhauser, M., Ding, Y. S., Hahn, A., Kroll, T., . . . Lanzenberger, R. (2012). Normative database of the serotonergic system in healthy subjects using multi-tracer PET. *Neuroimage, 63*(1), 447-459. doi:10.1016/j.neuroimage.2012.07.001

Sheehan, D. V., Lecrubier, Y., Sheehan, K. H., Amorim, P., Janavs, J., Weiller, E., . . . Dunbar, G. C. (1998). The Mini-International Neuropsychiatric Interview (M.I.N.I.): the development and validation of a structured diagnostic psychiatric interview for DSM-IV and ICD-10. *J Clin Psychiatry, 59 Suppl 20*, 22-33;quiz 34-57.

Smart, K., Cox, S. M. L., Scala, S. G., Tippler, M., Jaworska, N., Boivin, M., . . . Leyton, M. (2019). Sex differences in [(11)C]ABP688 binding: a positron emission tomography study of mGlu5 receptors. *Eur J Nucl Med Mol Imaging, 46*(5), 1179-1183. doi:10.1007/s00259-018-4252-4

Smith, S. M. (2002). Fast robust automated brain extraction. *Human Brain Mapping, 17*, 143-155.

Smith, S. M., Jenkinson, M., Woolrich, M. W., Beckmann, C. F., Behrens, T. E., Johansen-Berg, H., . . . Matthews, P. M. (2004). Advances in functional and structural MR image analysis and implementation as FSL. *Neuroimage, 23 Suppl 1*, S208-219. doi:10.1016/j.neuroimage.2004.07.051

Song, Y., Wang, C., Cai, H., Chen, J., Liu, S., Zhu, J., & Yu, Y. (2023). Functional hierarchy of the angular gyrus and its underlying genetic architecture. *Hum Brain Mapp, 44*(7), 2815-2828. doi:10.1002/hbm.26247

Thomas, G. E. C., Zarkali, A., Ryten, M., Shmueli, K., Gil-Martinez, A. L., Leyland, L. A., . . . Weil, R. S. (2021). Regional brain iron and gene expression provide insights into neurodegeneration in Parkinson's disease. *Brain, 144*(6), 1787-1798. doi:10.1093/brain/awab084

Thompson, E. (2015). Hamilton Rating Scale for Anxiety (HAM-A). *Occup Med (Lond), 65*(7), 601. doi:10.1093/occmed/kqv054

Turtonen, O., Saarinen, A., Nummenmaa, L., Tuominen, L., Tikka, M., Armio, R. L., . . . Hietala, J. (2021). Adult Attachment System Links With Brain Mu Opioid Receptor Availability In Vivo. *Biol Psychiatry Cogn Neurosci Neuroimaging, 6*(3), 360-369. doi:10.1016/j.bpsc.2020.10.013

Williams, J. B. (1988). A structured interview guide for the Hamilton Depression Rating Scale. *Arch Gen Psychiatry, 45*(8), 742-747. doi:10.1001/archpsyc.1988.01800320058007

Yan, C. G., Wang, X. D., Zuo, X. N., & Zang, Y. F. (2016). DPABI: Data Processing & Analysis for (Resting-State) Brain Imaging. *Neuroinformatics, 14*(3), 339-351. doi:10.1007/s12021-016-9299-4

Zakiniaeiz, Y., Hillmer, A. T., Matuskey, D., Nabulsi, N., Ropchan, J., Mazure, C. M., . . . Cosgrove, K. P. (2019). Sex differences in amphetamine-induced dopamine release in the dorsolateral prefrontal cortex of tobacco smokers. *Neuropsychopharmacology, 44*(13), 2205-2211. doi:10.1038/s41386-019-0456-y
